# Supplementary material for: Synthetic modeling reveals HOXB genes are critical for the initiation and maintenance of human leukemia
Source: Nat Commun. 2019 Jul 2;10:2913. doi: 10.1038/s41467-019-10510-8 (PMC6606637; doi:10.1038/s41467-019-10510-8)
Supplement: Supplementary file 1 — Supplementary Information [file 41467_2019_10510_MOESM1_ESM.pdf]

SUPPLEMENTARY INFORMATION

for

Synthetic modeling reveals HOXB genes are critical for the initiation and maintenance of human leukemia

Manabu Kusakabe, Ann Chong Sun, Kateryna Tyshchenko, Rachel Wong, Aastha Nanda, Claire Shanna, Samuel Gusscott, Elizabeth A. Chavez, Alireza Lorzadeh, Alice Zhu, Ainsleigh Hill, Stacy Hung, Scott Brown, Artem Babaian, Xuehai Wang, Robert A. Holt, Christian Steidl, Aly Karsan, R. Keith Humphries, Connie J. Eaves, Martin Hirst & Andrew P. Weng

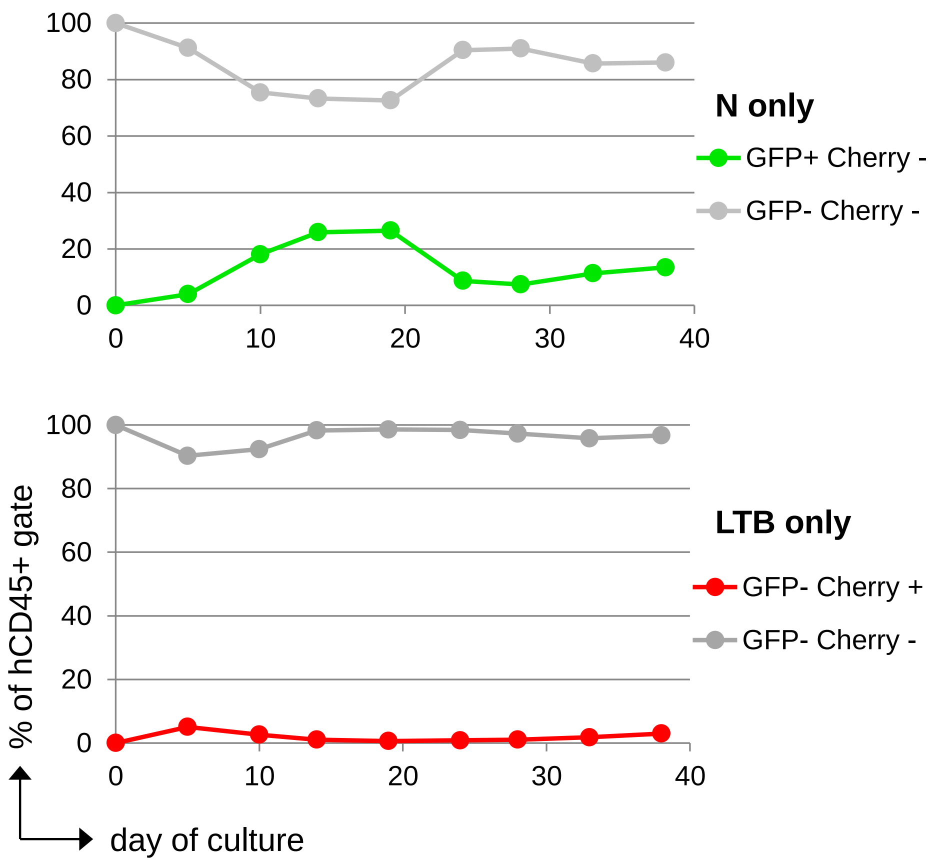

Supplementary Figure 1. Transduction with NOTCH1ΔE or LMO2-TAL1-BMI1 alone does not support CB expansion *in vitro*

Flow cytometric tracking of transduced CB cells. CD34+ CB cells were transduced with NOTCH1ΔE-GFP or LMO2-TAL1-BMI1-Cherry lentivirus on days 0 and 5, and cultured on OP9-DL1 feeders.

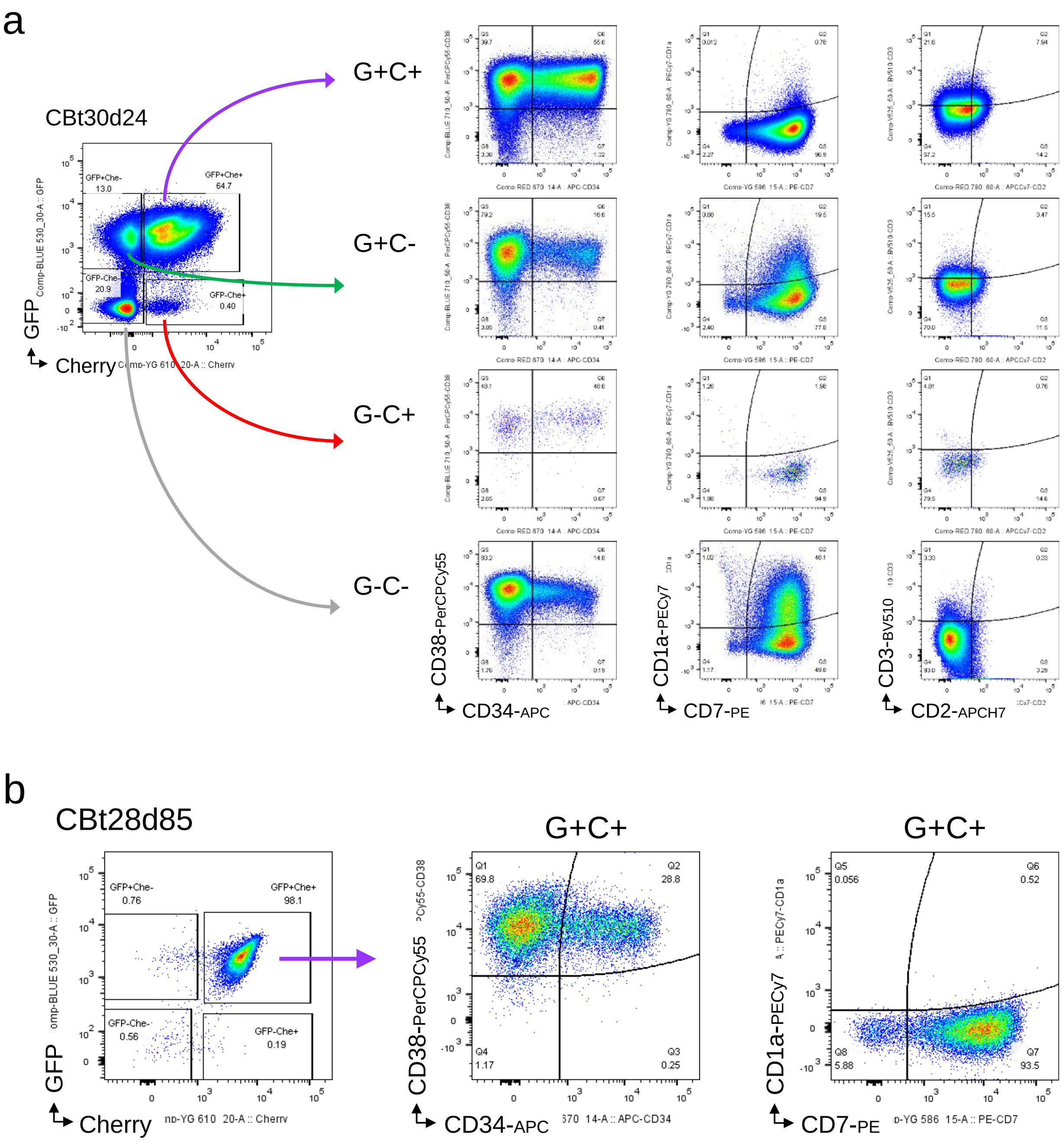

Supplementary Figure 2. Immunophenotype of transduced CB cells in OP9-DL1 co-cultures

Flow cytometric analysis. Representative plots of gated, live human CD45+ cells after **a)** 24 days and **b)** 85 days in culture.

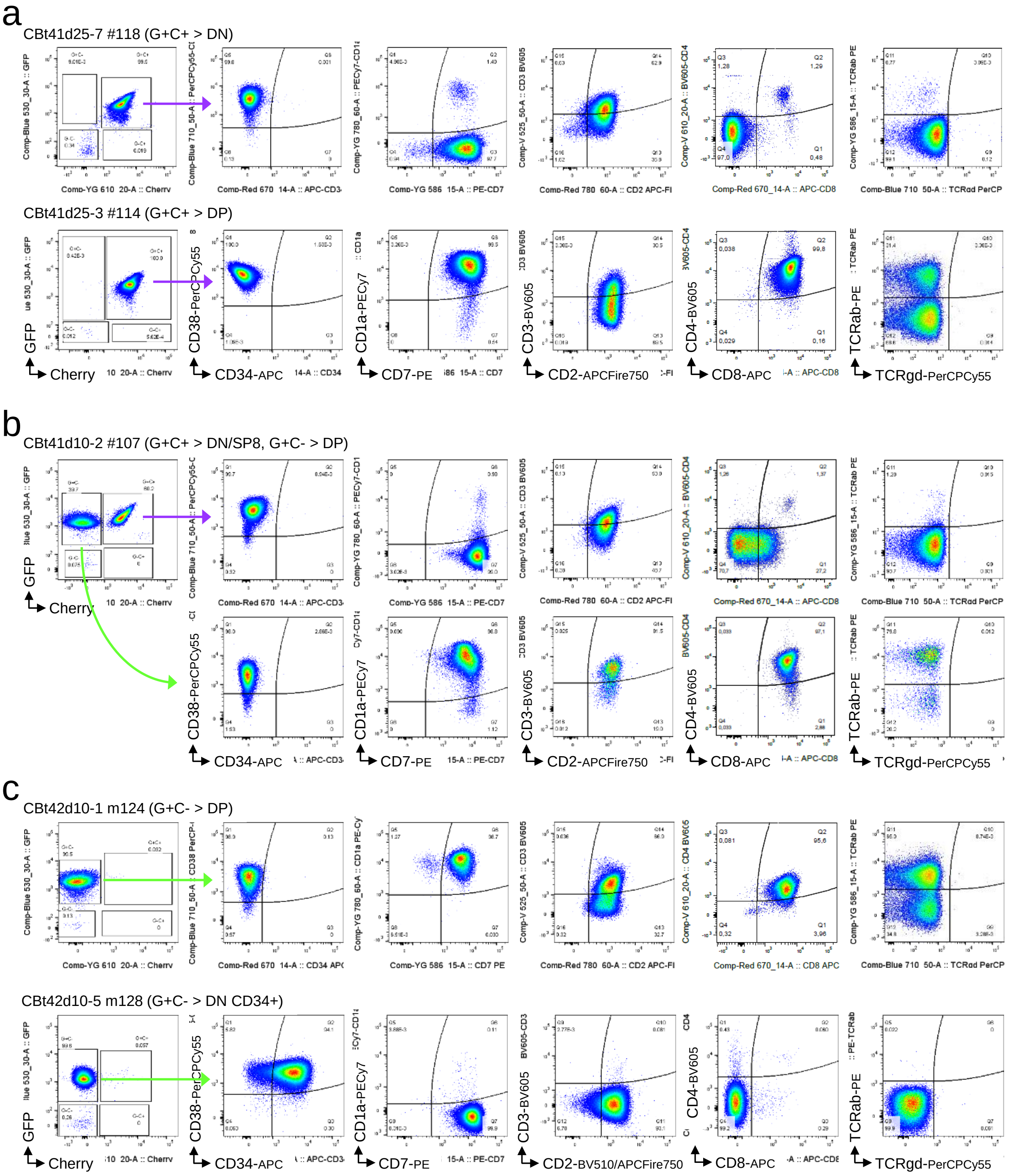

Supplementary Figure 3. Immunophenotype of CB leukemias from clinically morbid mice

Flow cytometric analysis. Representative plots of freshly explanted bone marrow cells from primary NSG recipients of transduced CB cells. All animals attained clinically morbid disease endpoints. Gated, live human CD45+ cells are shown. **a)** G+C+ leukemias. **b)** Mixed G+C+/G+C- leukemia. **c)** G+C- leukemias.

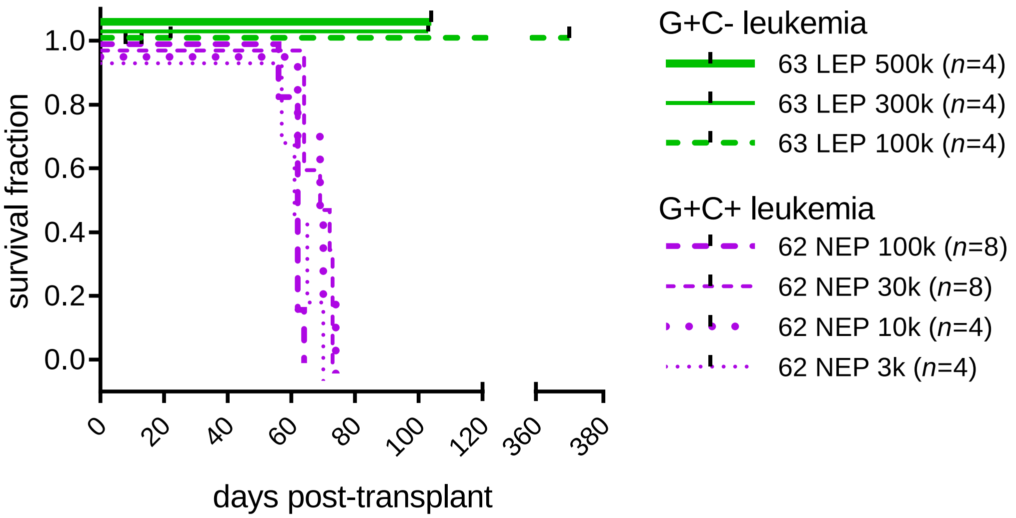

Supplementary Figure 4. Limiting dilution transplant analysis

Kaplan-Meier survival curves for secondary recipient mice transplanted with the indicated doses (500k to 3k) of primary NLTB-CB leukemia cells (CBt28d10-2 #62_NEP; G+C+) or N only-CB leukemia cells (CBt28d10-3 #63_LEP; G+C-). The number of recipients (n) for each cell dose are shown in parentheses. All leukemic animals achieved clinically morbid disease endpoints requiring euthanasia.

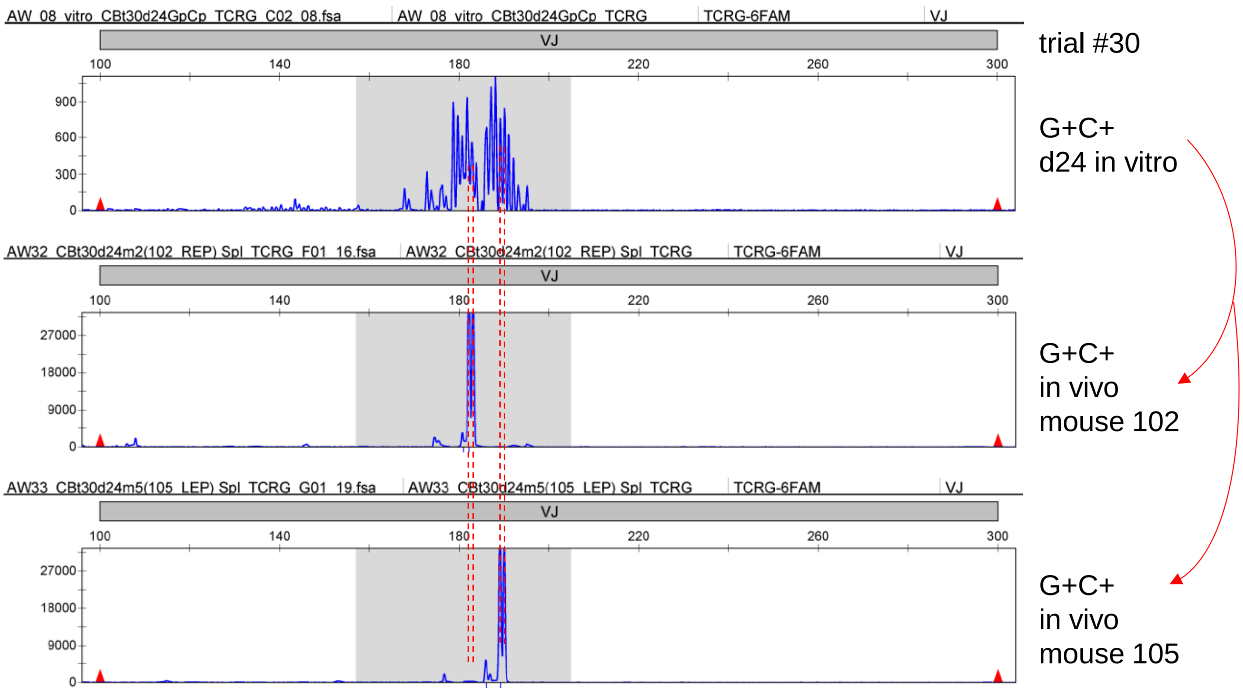

Supplementary Figure 5. BIOMED-2 TCRG clonality assay

Doubly transduced (G+C+) CB cells were FACS sorted from day 24 *in vitro* cultures, as well as from the leukemic mice into which the cultured cells were injected. Genomic DNA was then extracted, and the distribution of amplified TCRG DNA fragments analyzed bv GeneScan (Applied Biosystems). Dotted red lines are overlaid to facilitate comparison of peak sizes between samples. Peak sizes are reproducible within less than 0.5 bp.

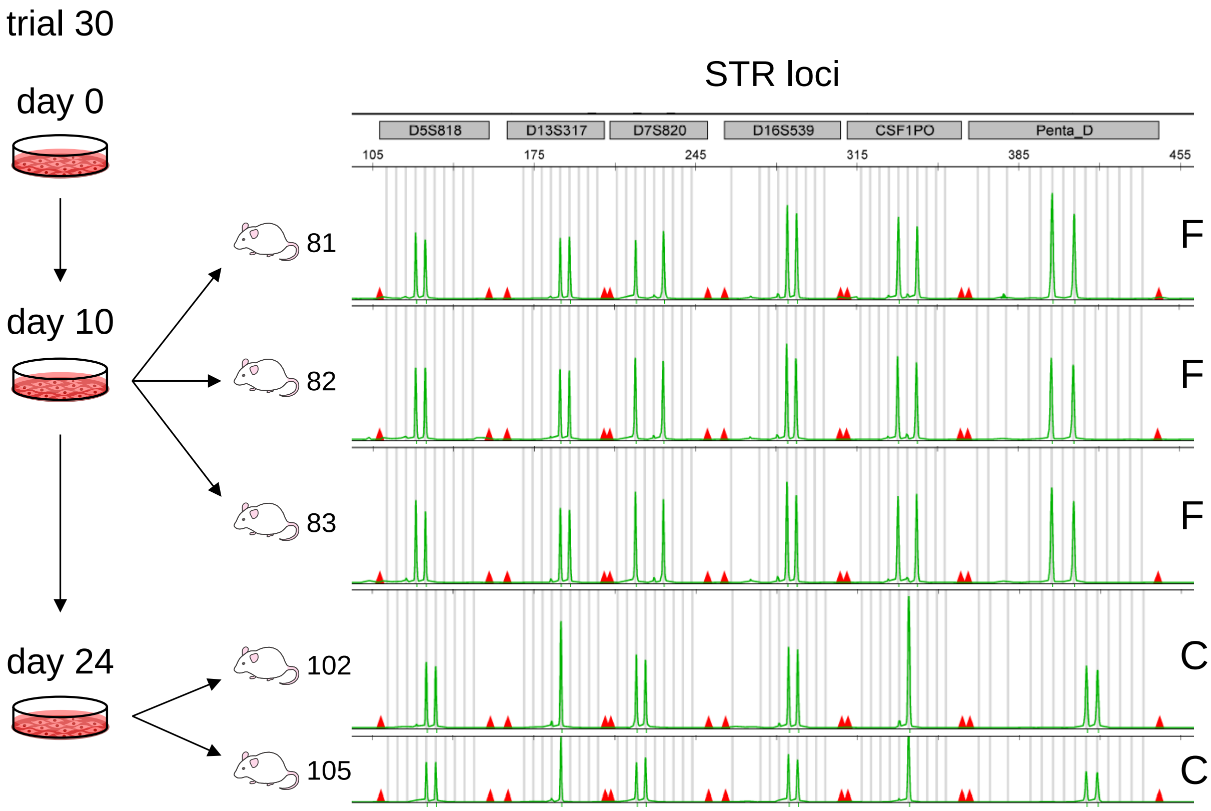

Supplementary Figure 6. STR profiling of CB leukemias

Genomic DNAs from primary CB leukemias were profiled by Promega PowerPlex 16HS assay. STR patterns from 2 different individual donors (C, F) can be discerned. The same pool of donor cord blood cells was used for trials #28 and #30.

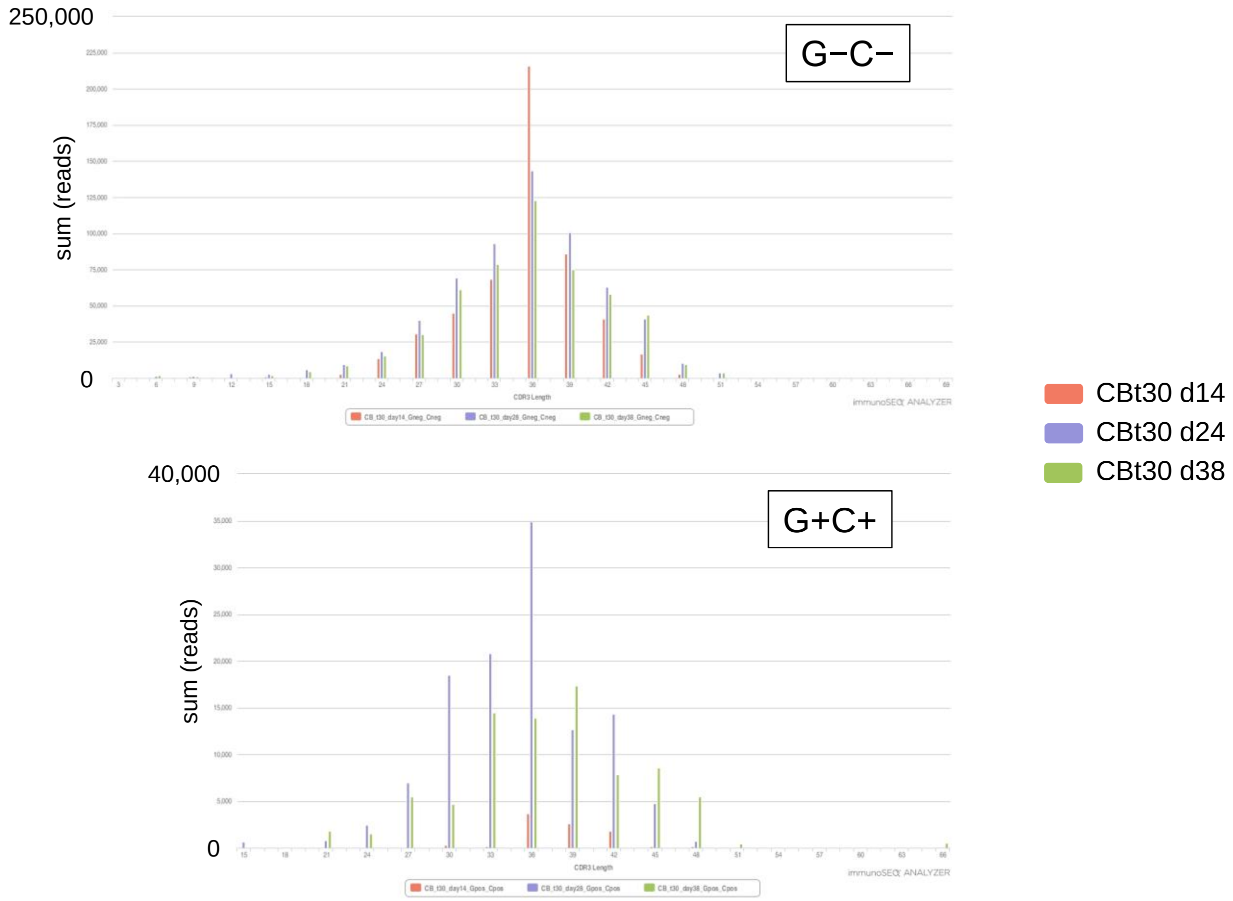

Supplementary Figure 7. TCRG CDR3 fragment length analysis from ImmunoSEQ data

ImmunoSEQ TCRG clonality assay. CB cells were transduced with N+LTB lentiviruses and cultured on OP9-DL1 feeders. Culture samples were taken at 14, 24, and 38 days. Doubly transduced (G+C+) and non-transduced (G-C-) subsets were FACS sorted and genomic DNA extracted for ImmunoSEQ TCRG (Survey) analysis by Adaptive Biotechnologies. Distributions of CDR3 fragment lengths are plotted.

Supplementary Figure 8. Variation among synthetic CB leukemias is mostly donor-related

a

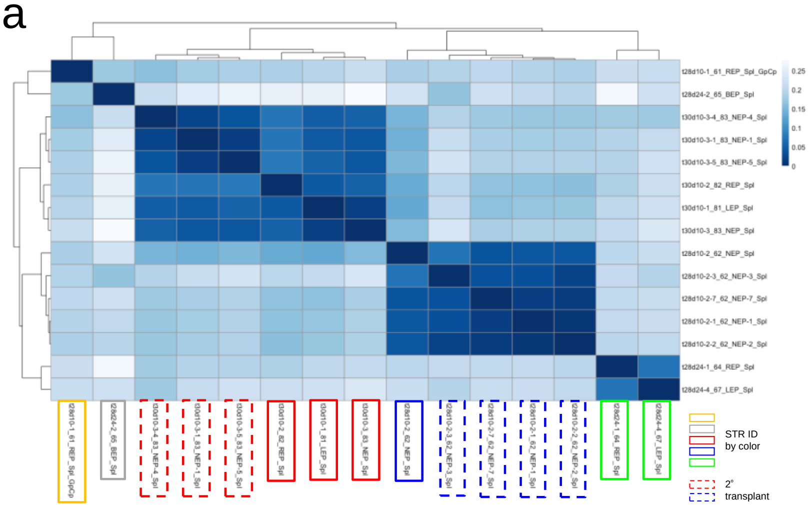

a) Unsupervised hierarchical clustering of 15 synthetic CB leukemias (NLTB G+C+) based on correlation distances (1-Spearman coefficient) from RNA-seq data. Correlation distances were calculated using the top 1,000 variable genes. Color scale is (1-Spearman) correlation distance. Darker blue indicates greater similarity.

b

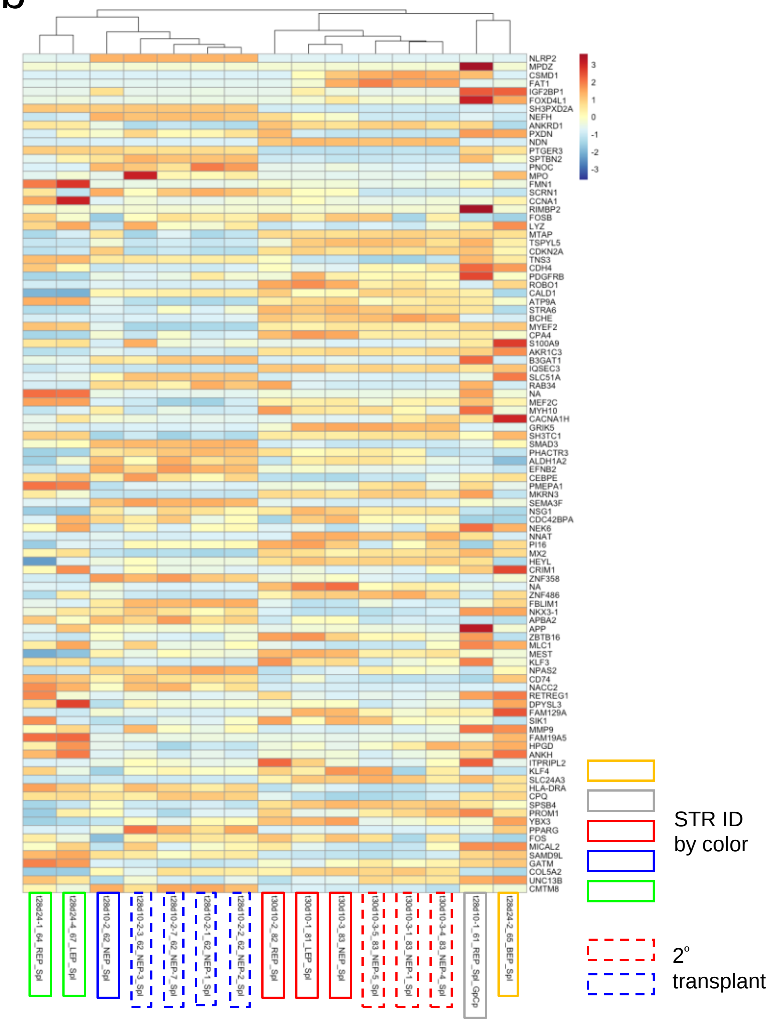

b) Unsupervised hierarchical clustering of 15 synthetic CB leukemias (NLTB G+C+) based on mRNA expression values from RNA-seq data. The top 100 variable protein coding genes (X/Y chromosomes excluded) were used for clustering. Genes are ordered top to bottom from most to least variation across the included samples. Color heatmap is scaled by gene (=row) with mean=0 and SD=1.

Individual CB donors were discriminated by STR profiling.

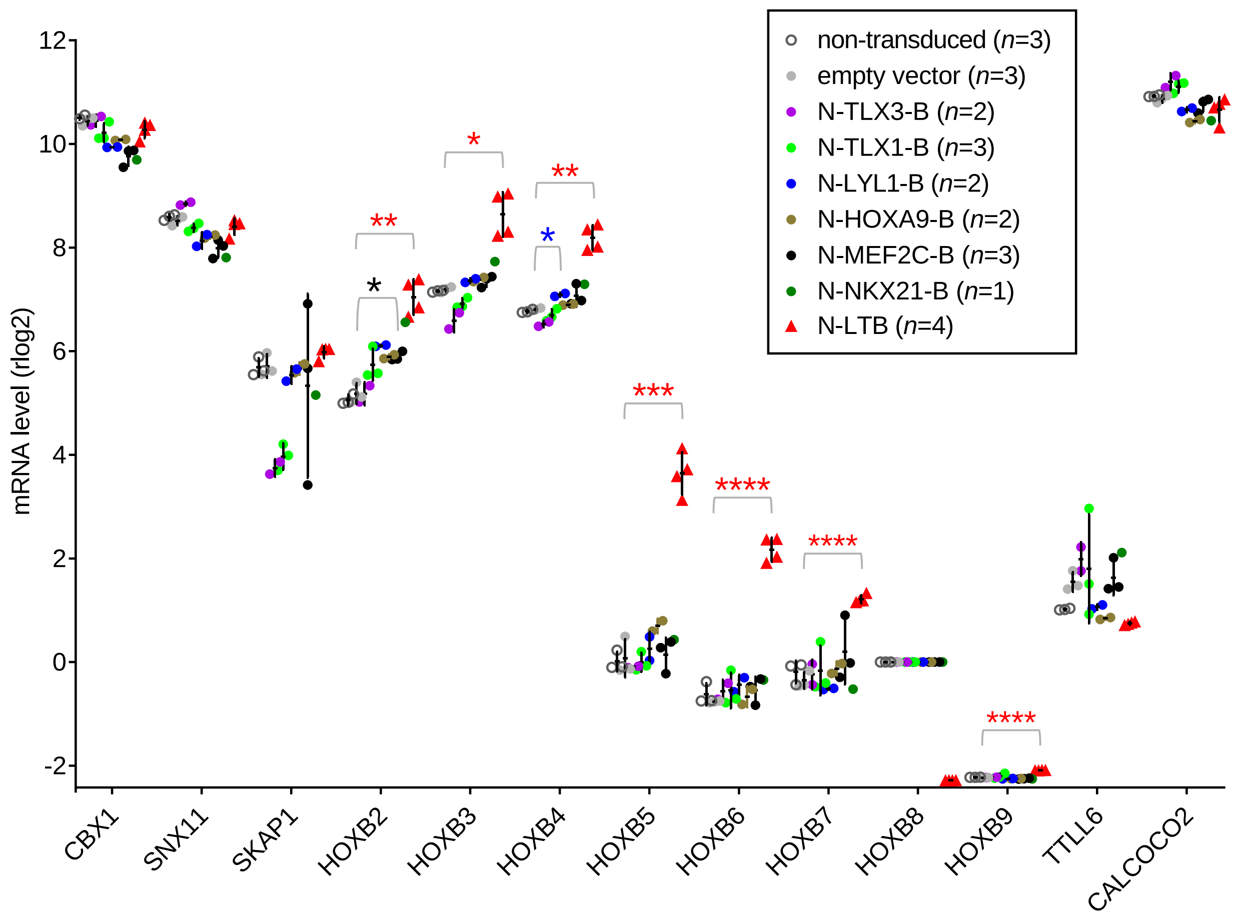

Supplementary Figure 9. Upregulation of multiple anterior HOXB genes is unique to the N+LTB combination of T-cell oncogenes

mRNA expression level of HOXB and flanking genes from RNA-seq of nascently transduced CB cells. CB cells were transduced with the indicated combinations of GFP and Cherry lentiviruses, then cultured on OP9-DL1 feeders for 19 days (except 2 samples for each of N-TLX1-B and N-MEF2C-B were collected after 52 days in order to obtain sufficient cells for library construction). RNA was prepared from FACS-sorted doubly (G+C+) transduced cells. Rlog values from DESeq2 are plotted. Each data point represents an individual sample. Bars indicate mean+/-SD for each set of colored dots. Statistical comparisons are shown only for significantly increased expression of HOXB genes as compared to empty vector-transduced controls.

**, p<0.05; **, p<0.01; ***, p<0.001 (one-tailed t-test with Holm-Sidak correction for multiple comparisons).*

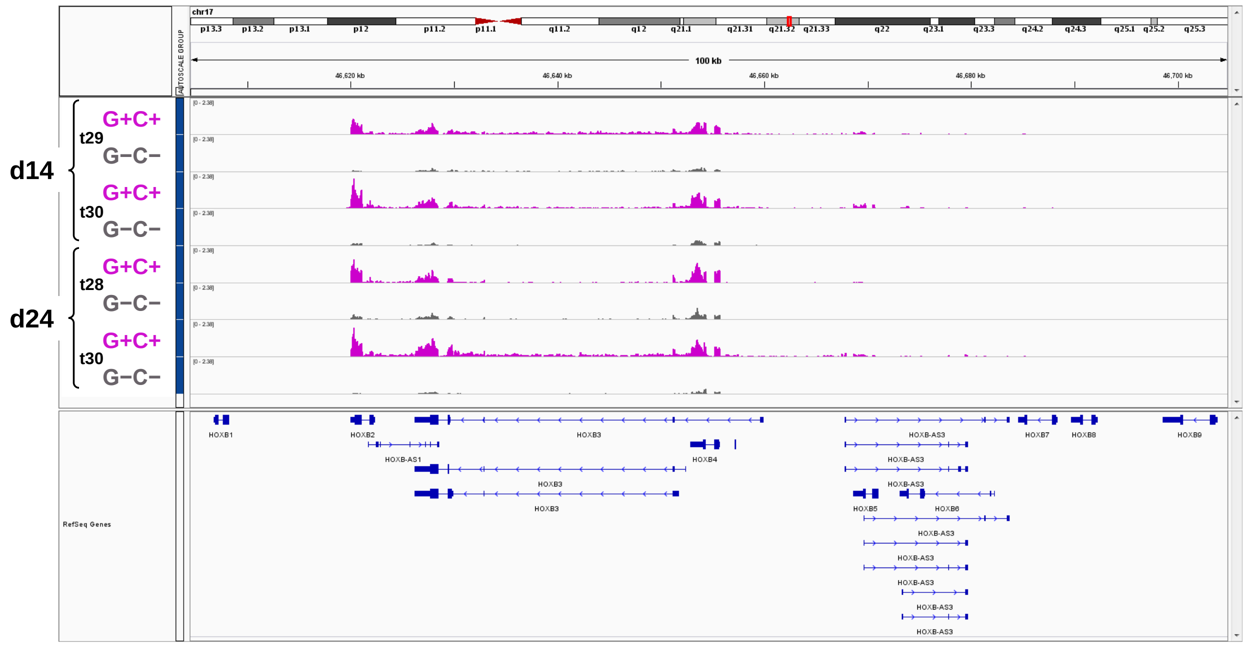

Supplementary Figure 10. RNA-seq tracks over the HOXB locus from NLTB-transduced CB cells

Individual RNA-seq tracks over the HOXB locus from FACS sorted, NLTB-transduced (G+C+) and non-transduced (G−C−) CB cells cultured for 14 and 24 days on OP9-DL1 feedersTrack heights are autoscaled according to the maximum sample with coverage values normalized per 1 million features.

d, day; t, experimental trial #.

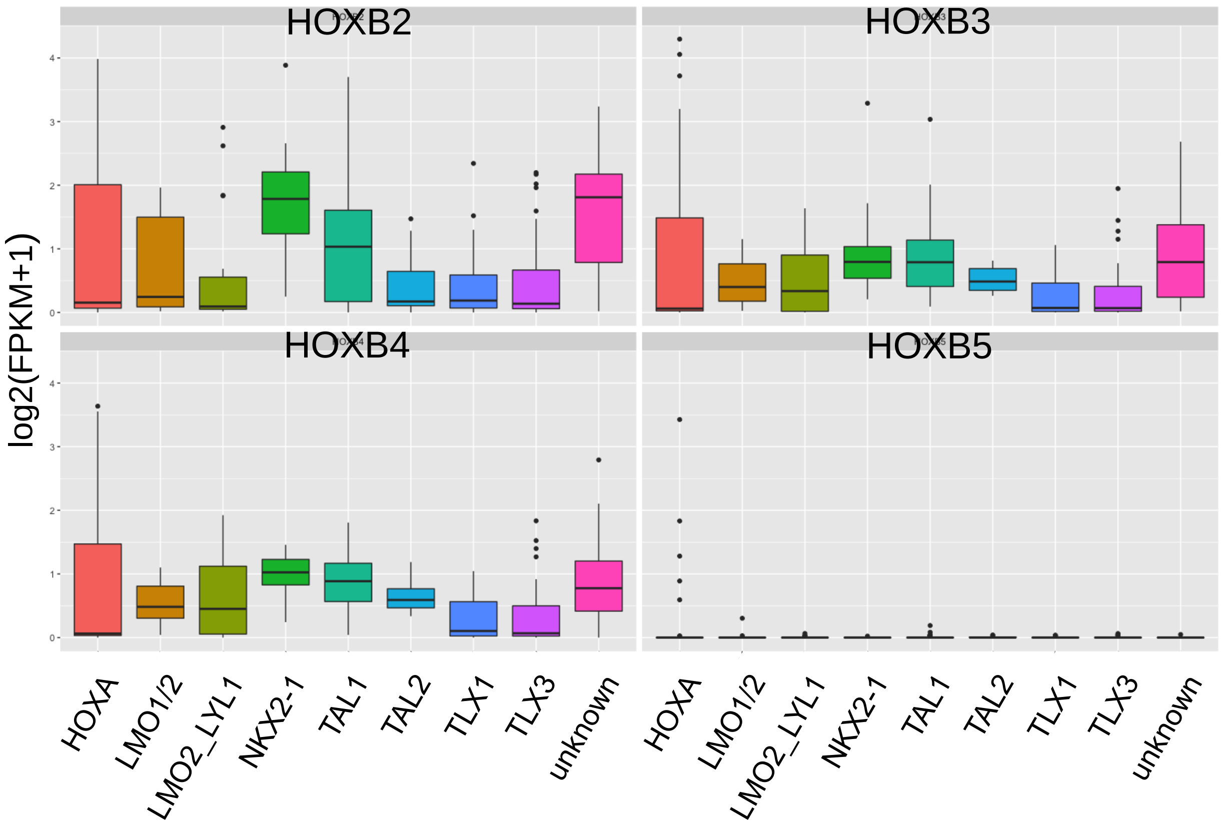

Supplementary Figure 11. HOXB gene expression levels across genetic/transcription factor subgroups of patient T-ALLs

HOXB gene expression levels from patient T-ALL RNA-seq data (252 patients from the COG TARGET study). Tukey box and whisker plots of log2(FPKM+1) values are shown. Genetic/transcription factor subgroups are as defined by Liu et al[1].

Data are reanalyzed from NCBI dbGaP accession phs000218/000464[1].

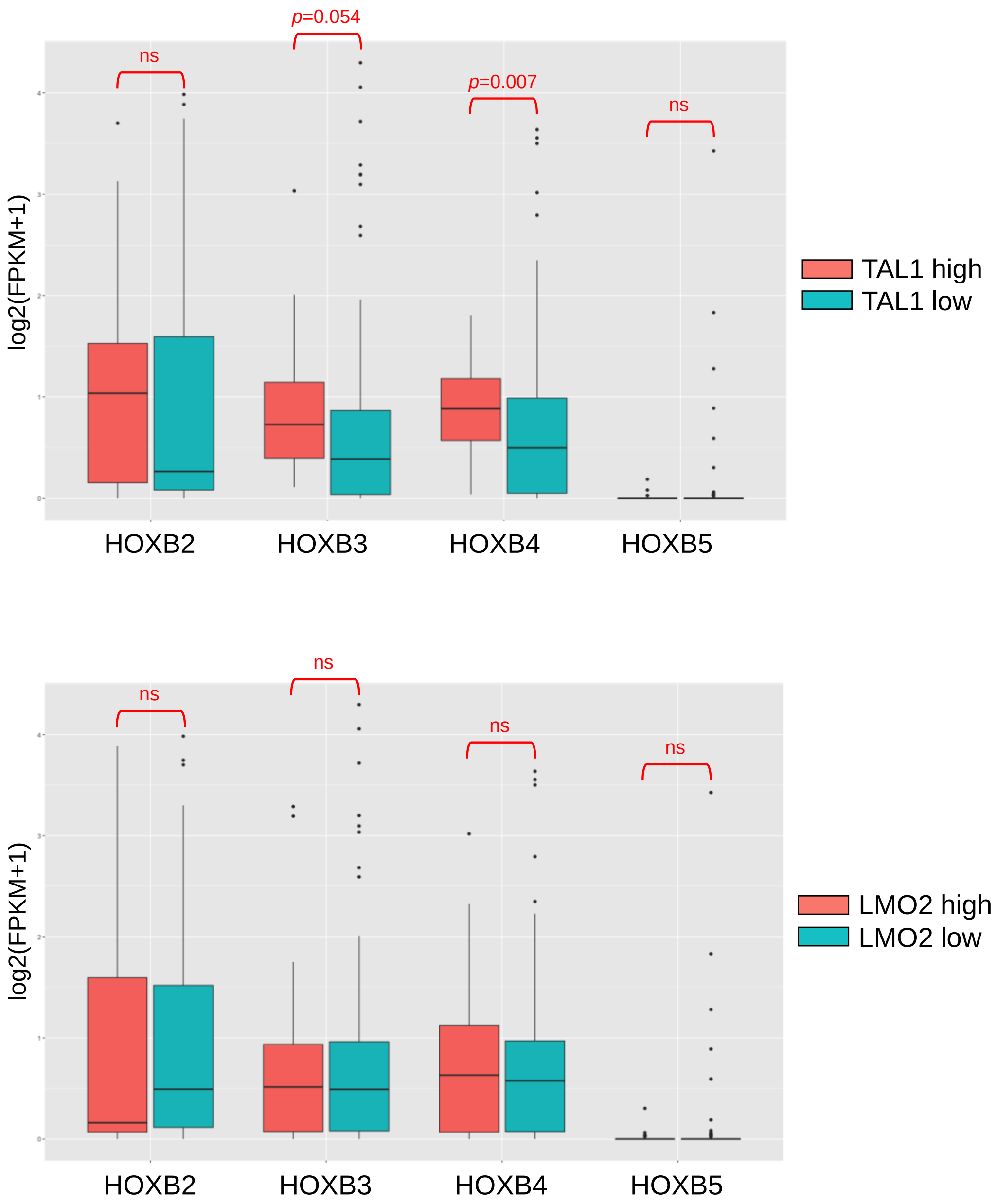

Supplementary Figure 12. HOXB gene expression levels among high vs low expressing subsets for TAL1 and LMO2

HOXB gene expression levels from patient T-ALL RNA-seq data (252 patients from the COG TARGET study). Tukey box and whisker plots of log2(FPKM+1) values are shown. The high expressing subsets were defined as the top quintile of expressers for the indicated gene, with the remainder included in the low group.

Statistical *p*-values are shown for comparisons between high vs. low subgroups (Welch's two-tailed *t*-test). *ns, not significant.*

Data are reanalyzed from NCBI dbGaP accession phs000218/000464[1].

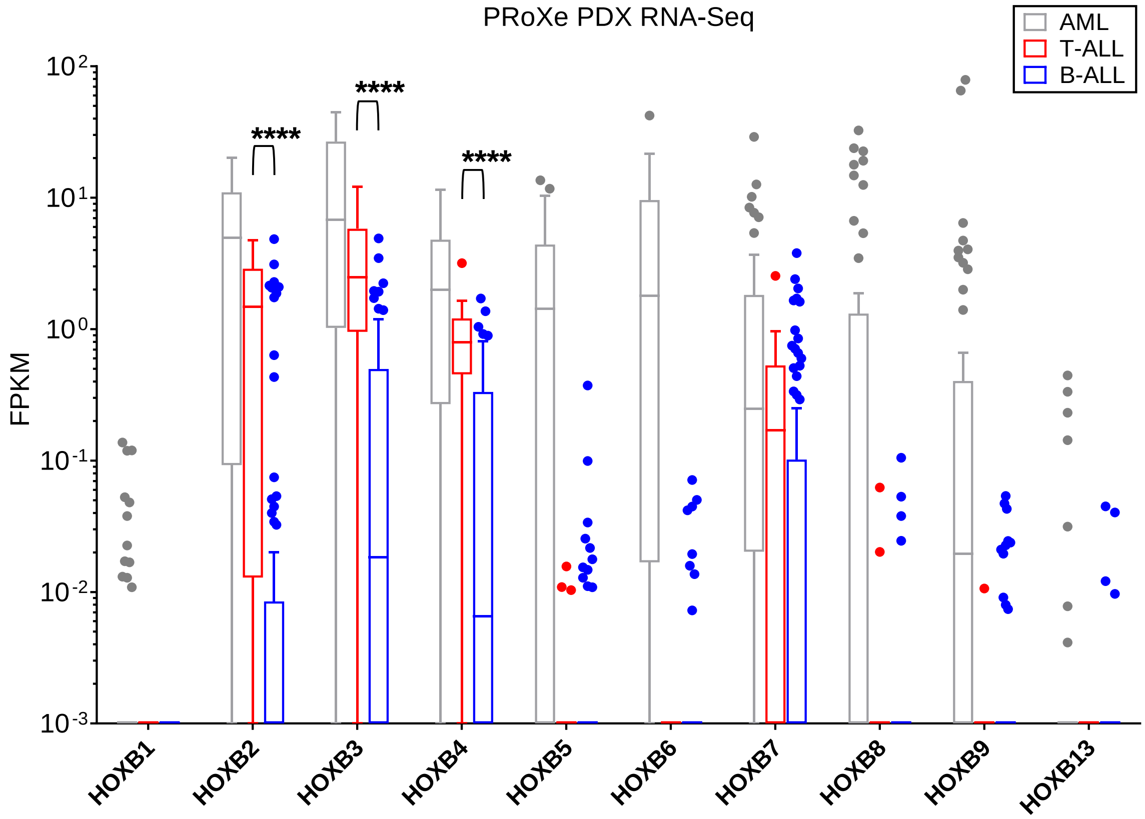

Supplementary Figure 13. HOXB gene expression patterns among different types of human acute leukemia

HOXB mRNA expression level in human T-ALL (*n*=34), B-ALL (*n*=111), and AML (*n*=54) PDX samples from the PRoXe repository[2]. FPKM values were calculated from RNA-seq data using Cufflinks. Tukey box and whiskers are shown (Boxes extend from 25th to 75th percentile. Horizontal line indicates the median. Whiskers indicate 1.5X the interquartile range above and below the box hinges, or the largest and smallest values, respectively. Datapoints beyond the whiskers are plotted individually).

Statistical *p*-values are shown only for comparisons between T-ALL and B-ALL. *****, p-adj <0.0001 (two-tailed t-test with Holm-Sidak correction for multiple comparisons). All other T-ALL vs. B-ALL comparisons are not significant.*

Data are reanalyzed from NCBI SRA accession SRP103099[2].

a

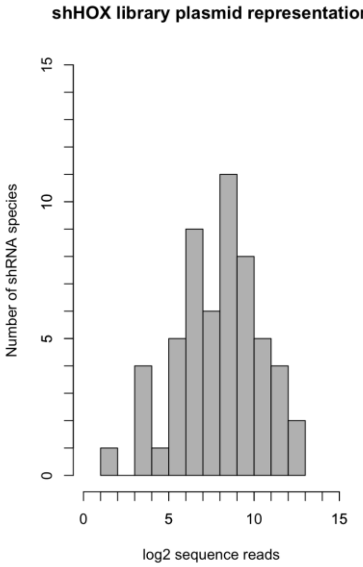

Supplementary Figure 14. Next generation sequencing of pooled shRNA library to assess representation of input clones

Histograms of clone representation.

a) Original pooled shRNA library at the plasmid DNA stage before production of lentivirus. The total number of identified shRNA species is 56, demonstrating that all input clones targeting HOX genes were recovered.

b) PCR amplified clones from genomic DNA collected 2 days after lentiviral transduction into cells. In each plot, the total number of identified shRNA species is 59, demonstrating that all input clones targeting HOX genes plus 3 controls were recovered.

b

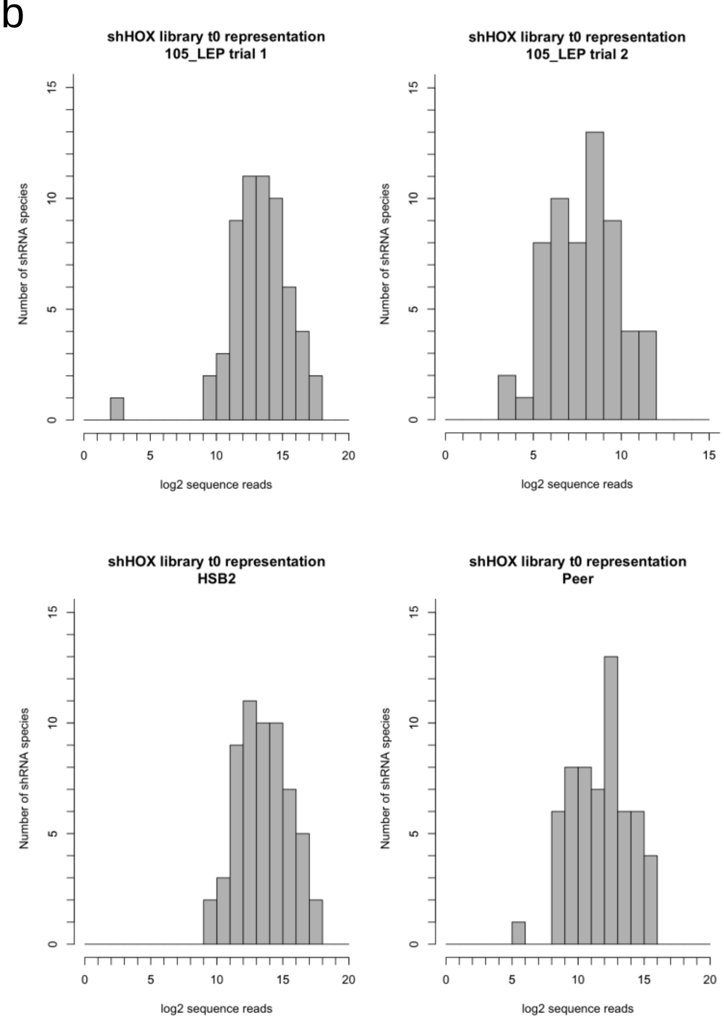

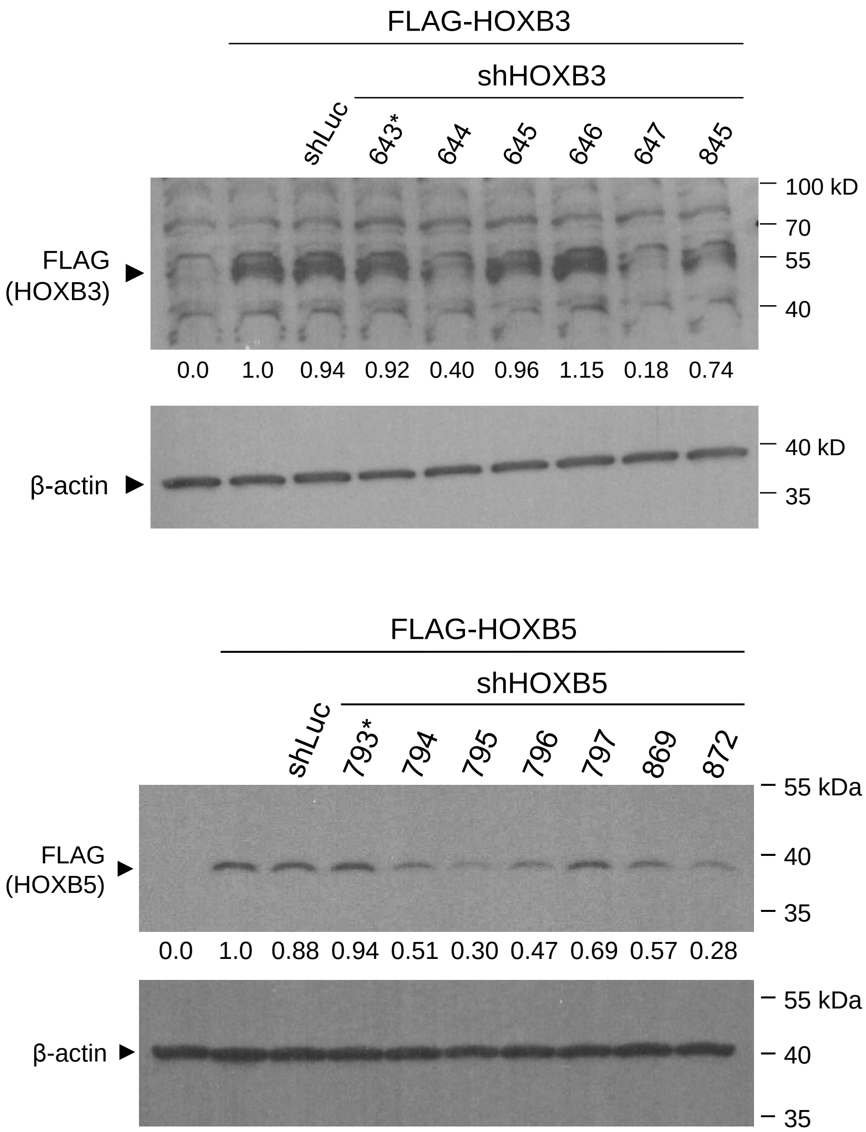

Supplementary Figure 15. Western blot validation of HOXB3 and HOXB5 knock-down

HOXB3 and HOXB5 protein levels by western blot assay. The human T-ALL cell line PF382 was first transduced with lentiviral FLAG-tagged HOXB3 or HOXB5 cDNA expression constructs. Transduced cells were FACS sorted (per blue fluorescent protein viral marker), and subsequently transduced with shRNA lentiviruses targeting HOXB3, HOXB5, or luciferase control (shLuc) with puromycin-resistance marker. shRNA-transduced cultures were selected with puromycin (1 ug ml^{-1}) for 3 days starting at day 2 post-transduction. Whole cell lysates were prepared and used for western blotting with antibodies as indicated. Numbers below each panel indicate band intensities after normalization to the β-actin loading control and are expressed relative to cells not transduced with shRNA virus. Full TRC designations for shRNA clones are provided in **Supplementary Table 6**.

*shHOXB3_643 and shHOXB5_793 clones target 3’ UTRs which were not present in the FLAG-tagged cDNA expression constructs.

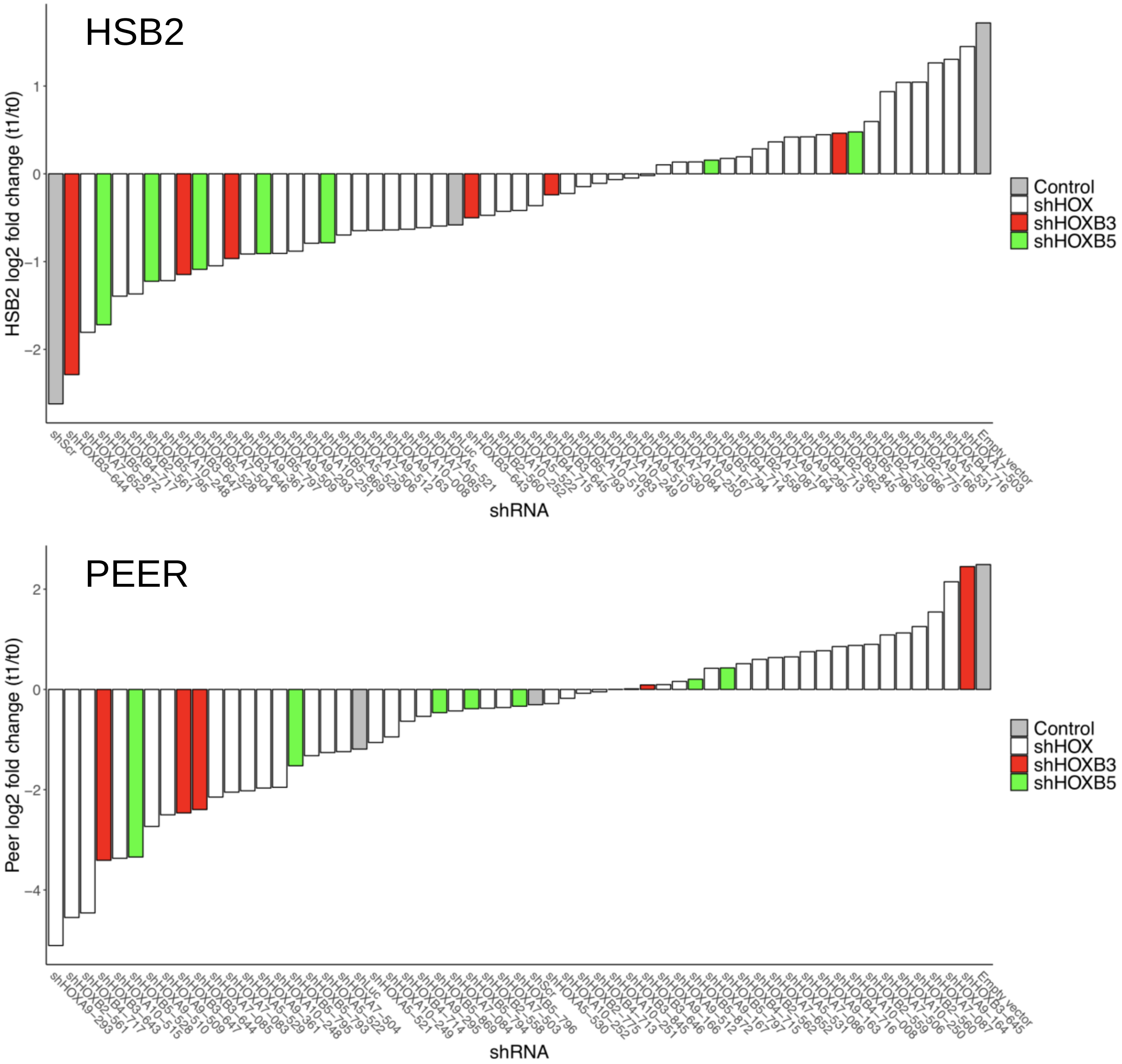

Supplementary Figure 16. Pooled HOX shRNA screen performed in established human T-ALL cell lines

Bar plot of HOX shRNA depletion/enrichment. Human T-ALL cell lines HSB2 and PEER were transduced with a pool of 56 lentiviral shRNAs targeting HOX genes (B2-B5, A5, A7, A9, A10) plus 3 non-targeting controls. Cells were harvested on days 2 (t0) and 9 (t1) post-transduction and genomic DNA extracted. Proviral shRNAs were enumerated by PCR amplification and NGS. The experiment was performed once in each of the two cell lines.

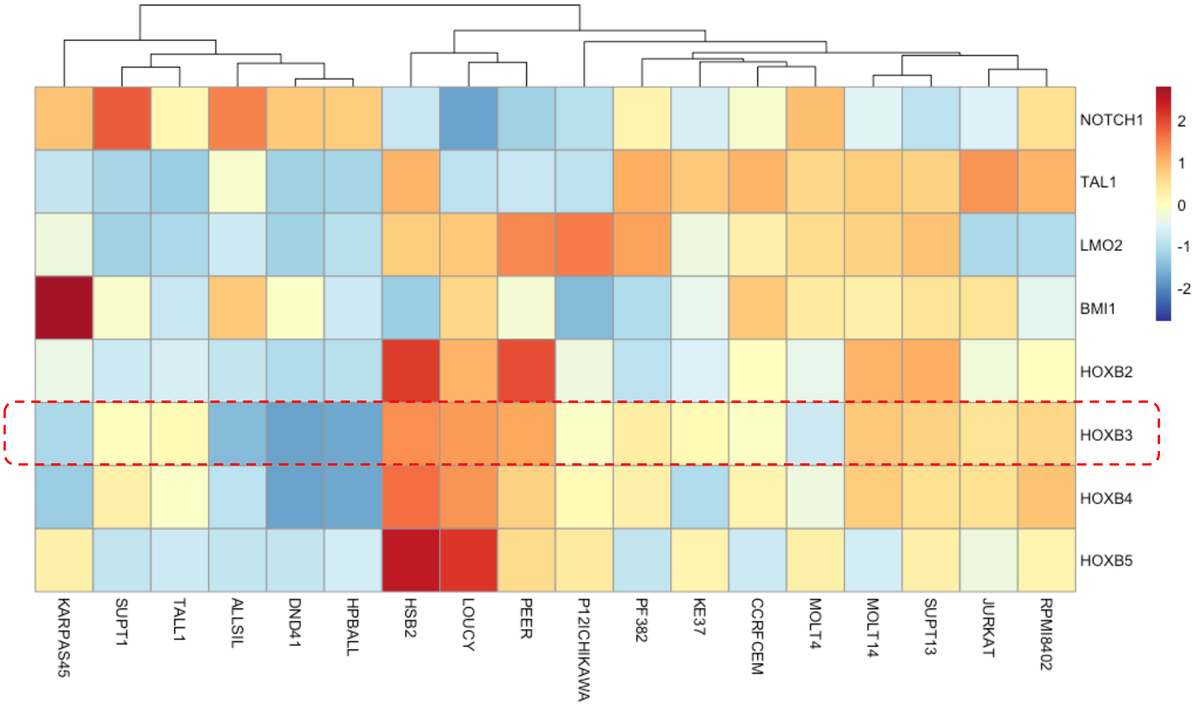

Supplementary Figure 17. NLTB and HOXB mRNA expression levels in established human T-ALL cell lines

mRNA expression heatmap. Rlog values were calculated from RNA-seq data using DESeq2. Heatmap is scaled by gene (=row) with mean=0 and SD=1.

Data are reanalyzed from EGA accession EGAD00001000849[3].

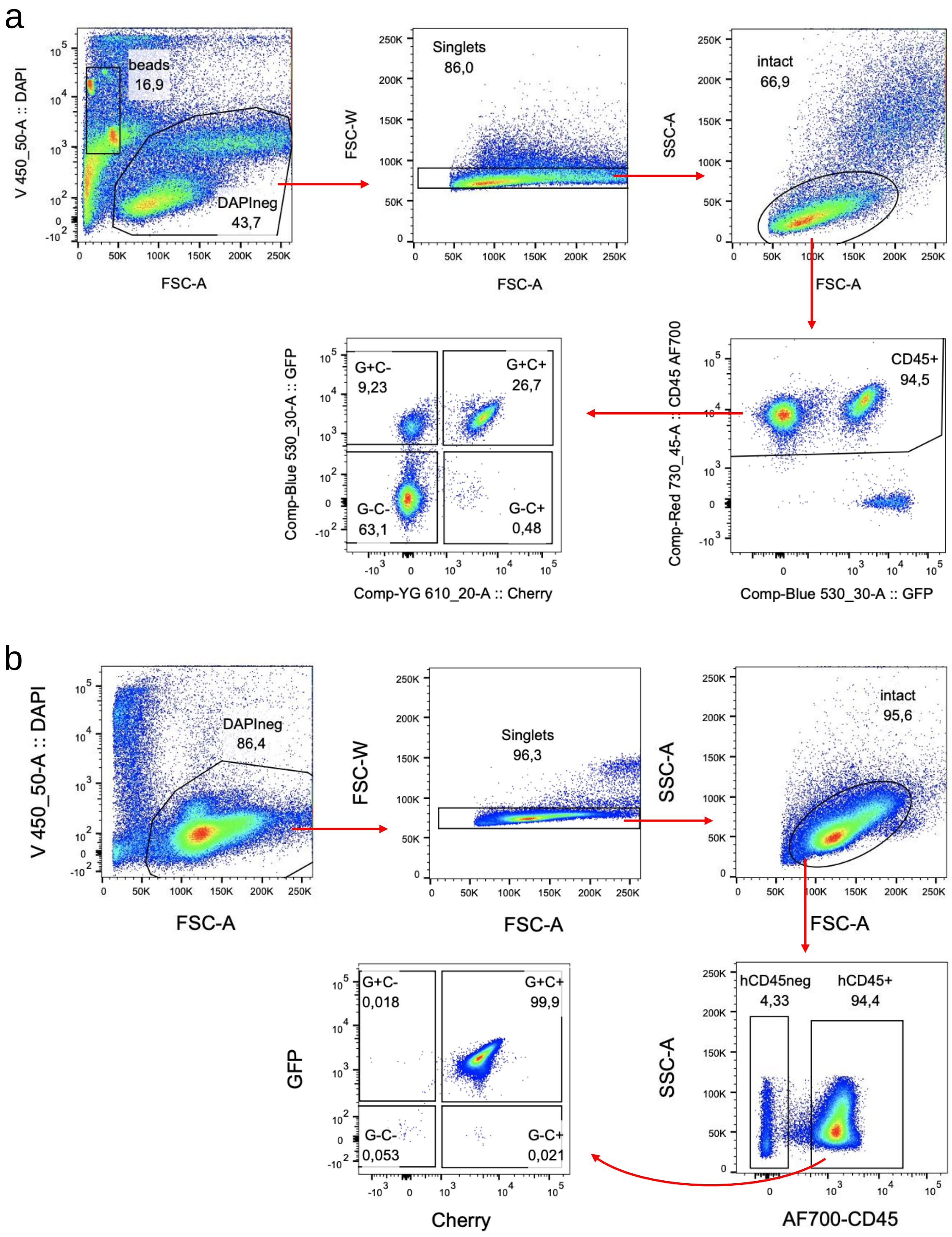

Supplementary Figure 18. Gating strategies used for flow cytometry analysis and sorting

Gating used to analyze/sort cells from **a)** *in vitro* cultures presented in Figs 1b, 1c, 1d, 1e, 3a, 3d, and 5a-d, and Supplementary Figs 1, 2, 7, 9, and 10; and **b)** *in vivo* samples presented in Supplementary Fig 3.

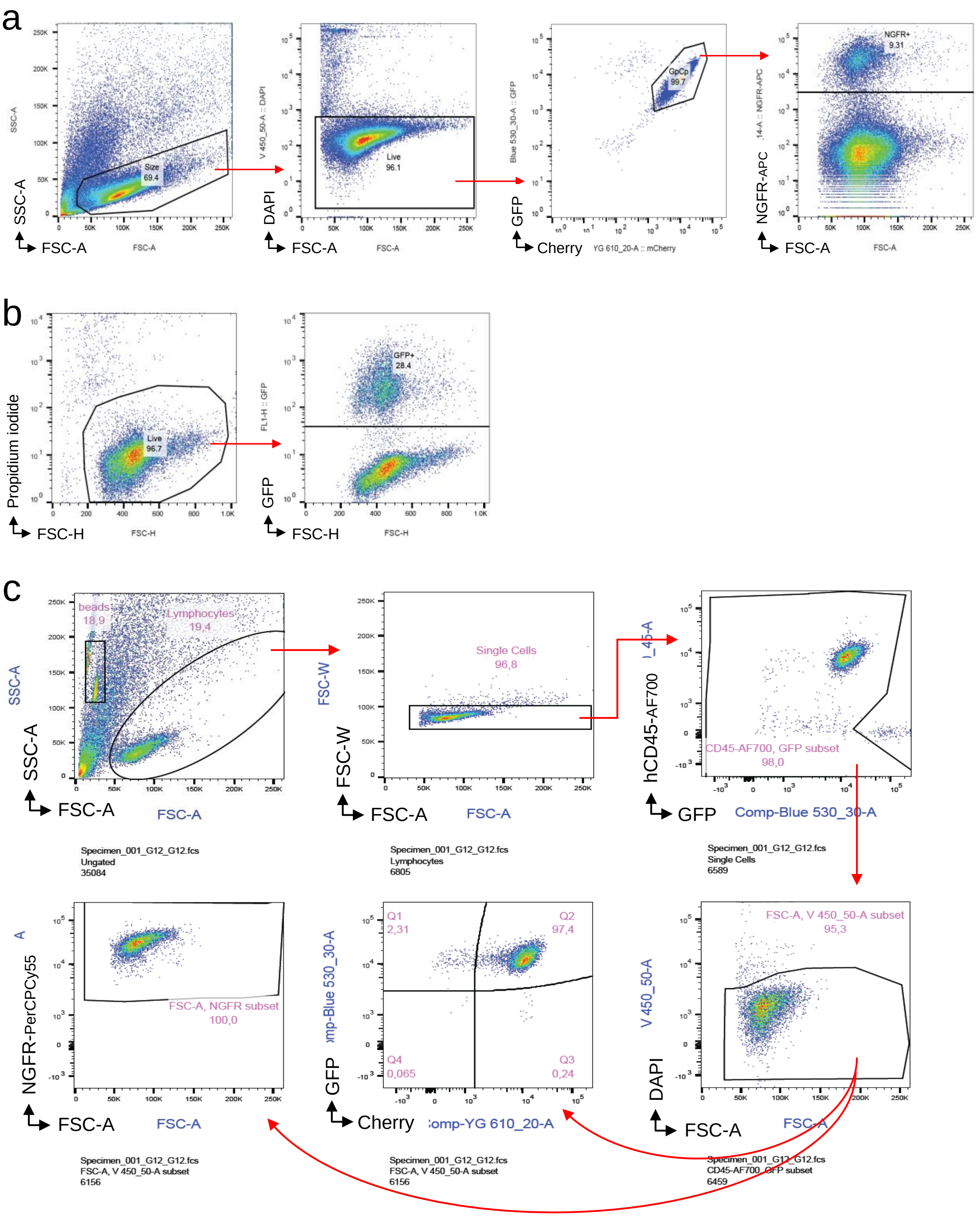

Supplementary Figure 19. Gating strategies used for flow cytometry analysis

Gating used to analyze cells from *in vitro* cultures presented in **a)** Fig 8c, **b)** Fig 8d, and **c)** Fig 8e.

Supplementary Table 1. Reactome pathway analysis – genes upregulated in NLTB-transduced CB cells

Analysis was performed with the list of 243 genes upregulated in NLTB (G+C+) vs. non-transduced (G-C-) CB cells as depicted in **Fig 5a**. Entries are ordered by increasing FDR. Pathways related to HOX genes are highlighted in red bold text.

Pathway ID	Pathway name	p-Value (<0.01)	FDR	Submitted entities found
R-HSA-9013508	NOTCH3 Intracellular Domain Regulates Transcription	1.21E-06	8.10E-04	HEYL;WWC1;HEY2;HES1
R-HSA-9012852	Signaling by NOTCH3	9.25E-06	0.0031	HEYL;WWC1;HEY2;HES1;EGFR
R-HSA-9013695	NOTCH4 Intracellular Domain Regulates Transcription	2.16E-05	0.0048	FLT4;HEY2;HES1
R-HSA-5619507	**Activation of HOX genes during differentiation**	4.20E-05	0.0056	CNOT6;MAFB;HOXB4;HOXB3;HOXB2
R-HSA-5617472	**Activation of anterior HOX genes in hindbrain development during early embryogenesis**	4.20E-05	0.0056	CNOT6;MAFB;HOXB4;HOXB3;HOXB2
R-HSA-194313	VEGF ligand-receptor interactions	6.82E-04	0.0598	FLT1;FLT4;VEGFA
R-HSA-195399	VEGF binds to VEGFR leading to receptor dimerization	6.82E-04	0.0598	FLT1;FLT4;VEGFA
R-HSA-1433557	Signaling by SCF-KIT	7.21E-04	0.0598	STAT5A;STAT3;PRKCA;PTPN6;MMP9;SH2B2
R-HSA-8866910	TFAP2 (AP-2) family regulates transcription of growth factors and their receptors	0.0011	0.0796	EGFR;VEGFA
R-HSA-2122947	NOTCH1 Intracellular Domain Regulates Transcription	0.0013	0.0847	HEYL;HEY2;HES1
R-HSA-3000171	Non-integrin membrane-ECM interactions	0.0020	0.1180	ITGB5;ITGA2;LAMC2;PRKCA;DMD;THBS1
R-HSA-9006934	Signaling by Receptor Tyrosine Kinases	0.0033	0.1271	STAT5A;VAV3;PRR5;MEF2C;FLT1;ITGA2;FLT4;STAT3;LAMC2;PRKCA;FURIN;MMP9;THBS1;EGFR;VEGFA;ADCYAP1;PTPN6;WASF1;TNS3;SH2B2
R-HSA-2894862	Constitutive Signaling by NOTCH1 HD+PEST Domain Mutants	0.0033	0.1271	HEYL;HEY2;HES1
R-HSA-2644606	Constitutive Signaling by NOTCH1 PEST Domain Mutants	0.0033	0.1271	HEYL;HEY2;HES1
R-HSA-2644602	Signaling by NOTCH1 PEST Domain Mutants in Cancer	0.0033	0.1271	HEYL;HEY2;HES1
R-HSA-2894858	Signaling by NOTCH1 HD+PEST Domain Mutants in Cancer	0.0033	0.1271	HEYL;HEY2;HES1
R-HSA-2644603	Signaling by NOTCH1 in Cancer	0.0033	0.1271	HEYL;HEY2;HES1
R-HSA-3000170	Syndecan interactions	0.0034	0.1271	ITGB5;ITGA2;PRKCA;THBS1
R-HSA-1234158	Regulation of gene expression by Hypoxia-inducible Factor	0.0040	0.1414	EPAS1;VEGFA
R-HSA-6785807	Interleukin-4 and Interleukin-13 signaling	0.0054	0.1711	STAT3;RHOU;RORA;PTGS2;MMP9;VEGFA
R-HSA-6804759	Regulation of TP53 Activity through Association with Co-factors	0.0057	0.1711	POU4F1;ZNF385A
R-HSA-5676594	TNF receptor superfamily (TNFSF) members mediating non-canonical NF-kB pathway	0.0057	0.1711	CD40;CD40LG;TNFRSF13C

R-HSA-1433559	Regulation of KIT signaling	0.0067	0.1800	PRKCA;PTPN6;SH2B2
R-HSA-417957	P2Y receptors	0.0067	0.1800	GPR17;P2RY11;P2RY2
R-HSA-452723	Transcriptional regulation of pluripotent stem cells	0.0074	0.1921	EPAS1;STAT3;DKK1
R-HSA-194138	Signaling by VEGF	0.0078	0.1958	VAV3;PRR5;FLT1;FLT4;PRKCA;WASF1;VEGFA
R-HSA-1980143	Signaling by NOTCH1	0.0086	0.1990	HEYL;HEY2;HES1
R-HSA-157118	Signaling by NOTCH	0.0087	0.1990	HEYL;FLT4;WWC1;HEY2;HES1;FURIN;EGFR
R-HSA-445355	Smooth Muscle Contraction	0.0096	0.2200	ITGB5;CALD1;ITGA1;SORBS1

Supplementary Table 2. Reactome pathway analysis – genes downregulated in NLTB-transduced CB cells

Analysis was performed with the list of 225 genes downregulated in NLTB (G+C+) vs. non-transduced (G-C-) CB cells as depicted in **Fig 5a**. Entries are ordered by increasing FDR.

Pathway ID	Pathway name	p-Value (<0.01)	FDR	Submitted entities found
R-HSA-168256	Immune System	2.38E-06	0.0014	FCN1;CSF3R;SERPINA1;MS4A3;FLT3;CLEC10A;RORC;EPX;MPO;GLIPR1;CDH1;BLNK;ITGAX;CD36;RAG2;TNFRSF4;CCR2;IL13RA1;RAG1;DUSP4;FCER1G;TNFRSF18;CYBB;RNASE2;NCR2;CLEC4C;JAML;CD8B;IL1B;CD300E;TLR7;PRTN3;SLC29A1;S100A8;SERPINB10;CAMK2D;CLEC12A;CEBPD;KLRB1;CD1C;DTX4;RASGRP1;CD1A;LILRA4;CST3;BTLA;SLAMF7;HLA-DQA1;IL32;MGAM;FYB1;SMAD3;PTPN13;S100B;LYZ;PTPN14;LILRB4;PTK2;CD4;IL2RA;MNDA;TNFSF8;PDCD1;CD247
R-HSA-449147	Signaling by Interleukins	5.47E-06	0.0017	IL32;DUSP4;CSF3R;SMAD3;CEBPD;FLT3;RORC;PTPN13;S100B;PTPN14;CD4;IL1B;IL2RA;BLNK;ITGAX;PRTN3;CD36;RAG2;CCR2;IL13RA1;RAG1
R-HSA-6798695	Neutrophil degranulation	2.31E-05	0.0046	SERPINB10;FCN1;MGAM;FCER1G;MS4A3;SERPINA1;CLEC12A;CYBB;RNASE2;EPX;LYZ;LILRB4;MPO;CST3;GLIPR1;CLEC4C;ITGAX;MNDA;PRTN3;CD36;SLC29A1;S100A8
R-HSA-449836	Other interleukin signaling	1.21E-04	0.0183	IL32;CD4;CSF3R;FLT3;PRTN3
R-HSA-1280215	Cytokine Signaling in Immune system	1.51E-04	0.0183	CAMK2D;CSF3R;CEBPD;FLT3;RORC;BLNK;ITGAX;CD36;RAG2;TNFRSF4;HLA-DQA1;CCR2;IL13RA1;RAG1;IL32;DUSP4;SMAD3;TNFRSF18;PTPN13;S100B;PTPN14;CD4;IL1B;IL2RA;TNFSF8;PRTN3
R-HSA-8877330	RUNX1 and FOXP3 control the development of regulatory T lymphocytes (Tregs)	2.78E-04	0.0278	IL2RA;TNFRSF18
R-HSA-5660668	CLEC7A/inflammasome pathway	4.39E-04	0.0377	IL1B
R-HSA-9008059	Interleukin-37 signaling	5.34E-04	0.0401	SMAD3;PTPN13;PTPN14
R-HSA-389948	PD-1 signaling	0.0014	0.0960	CD4;PDCD1;CD247;HLA-DQA1
R-HSA-6785807	Interleukin-4 and Interleukin-13 signaling	0.0017	0.1016	CEBPD;IL1B;RORC;ITGAX;CD36;IL13RA1
R-HSA-380108	Chemokine receptors bind chemokines	0.0019	0.1016	CX3CR1;CXCR4;CCR8;CCR2
R-HSA-168249	Innate Immune System	0.0020	0.1016	SERPINB10;FCN1;MS4A3;SERPINA1;CLEC12A;CLEC10A;EPX;DTX4;RASGRP1;MPO;CST3;GLIPR1;ITGAX;CD36;CCR2;DUSP4;MGAM;FCER1G;CYBB;LYZ;S100B;RNASE2;LILRB4;PTK2;NCR2;CD4;CLEC4C;IL1B;CD300E;MNDA;TLR7;PRTN3;CD247;SLC29A1;S100A8
R-HSA-5683057	MAPK family signaling cascades	0.0027	0.1249	DUSP4;CAMK2D;RASA3;FLT3;IL2RA;KIT;RASAL2;RAG2;DUSP16;RASGRP1;PTK2;RAG1
R-HSA-1475029	Reversible hydration of carbon dioxide	0.0037	0.1587	CA2;CA6
R-HSA-202433	Generation of second messenger molecules	0.0042	0.1587	CD4;FYB1;CD247;HLA-DQA1
R-HSA-9013508	NOTCH3 Intracellular Domain Regulates Transcription	0.0043	0.1587	PTCRA;PBX1

R-HSA-198933	Immunoregulatory interactions between a Lymphoid and a non-Lymphoid cell	0.0051	0.1709	NCR2;CD8B;JAML;KLRB1;CDH1;CD300E;SLAMF7;CD247;CD1C;LILRB4;CD1A;LILRA4
R-HSA-2129379	Molecules associated with elastic fibres	0.0052	0.1709	FBN2;MFAP4;BMP10;FBLN1
R-HSA-202430	Translocation of ZAP-70 to Immunological synapse	0.0073	0.2267	CD4;CD247;HLA-DQA1
R-HSA-388841	Costimulation by the CD28 family	0.0087	0.2589	CD4;BTLA;PDCD1;CD247;HLA-DQA1
R-HSA-202427	Phosphorylation of CD3 and TCR zeta chains	0.0092	0.2589	CD4;CD247;HLA-DQA1
R-HSA-1566948	Elastic fibre formation	0.0100	0.2689	FBN2;MFAP4;BMP10;FBLN1

Supplementary Table 3. Reactome pathway analysis – genes upregulated *in vivo*

Analysis was performed with the list of 31 genes upregulated in primary NLTB leukemia cells vs. NLTB-transduced CB cells cultured *in vitro*. Entries are ordered by increasing FDR.

Pathway ID	Pathway name	p-Value (<0.01)	FDR	Submitted entities found
R-HSA-9013508	NOTCH3 Intracellular Domain Regulates Transcription	1.88E-06	1.93E-04	PTCRA;HEY1
R-HSA-9012852	Signaling by NOTCH3	1.68E-05	8.56E-04	PTCRA;HEY1
R-HSA-9013695	NOTCH4 Intracellular Domain Regulates Transcription	0.0018	0.0627	HEY1
R-HSA-157118	Signaling by NOTCH	0.0033	0.0711	PTCRA;HEY1
R-HSA-6802948	Signaling by high-kinase activity BRAF mutants	0.0056	0.0711	KSR2
R-HSA-5674135	MAP2K and MAPK activation	0.0058	0.0711	KSR2
R-HSA-6802946	Signaling by moderate kinase activity BRAF mutants	0.0071	0.0711	KSR2
R-HSA-6802955	Paradoxical activation of RAF signaling by kinase inactive BRAF	0.0074	0.0711	KSR2
R-HSA-2122947	NOTCH1 Intracellular Domain Regulates Transcription	0.0082	0.0711	HEY1

Supplementary Table 4. Reactome pathway analysis – genes downregulated *in vivo*

Analysis was performed with the list of 96 genes downregulated in primary NLTB leukemia cells vs. NLTB-transduced CB cells cultured *in vitro*. Entries are ordered by increasing FDR.

Pathway ID	Pathway name	p-Value (<0.01)	FDR	Submitted entities found
**R-HSA-9012546**	Interleukin-18 signaling	5.18E-08	1.68E-05	ALOX5;IL18
**R-HSA-6783783**	Interleukin-10 signaling	8.97E-07	1.45E-04	CXCL8;IL1R1;IL18;PTGS2
**R-HSA-6785807**	Interleukin-4 and Interleukin-13 signaling	2.25E-06	2.20E-04	CXCL8;ALOX5;IL18;STAT4;RORA;PTGS2
**R-HSA-1280215**	Cytokine Signaling in Immune system	2.72E-06	2.20E-04	SOCS2;CXCL8;IL1R1;ALOX5;HLA-DPB1;IL18;STAT4;IRF5;RORA;SAMHD1;PTGS2;HLA-DPA1
**R-HSA-168256**	Immune System	1.02E-05	6.56E-04	CXCL8;CFH;RORA;SAMHD1;PTGS2;PLD1;SOCS2;HLA-DMA;ALOX5;STAT4;ATP6V0A4;CCR6;CD34;CD33;HLA-DPA1;SIGLEC12;IL1R1;SIGLEC10;IL18;COMMD3;SERPINB6;HLA-DPB1;BTK;IRF5;DOCK1;SLC27A2;CD200;SIGLEC6
**R-HSA-449147**	Signaling by Interleukins	3.31E-05	0.0018	SOCS2;CXCL8;IL1R1;ALOX5;IL18;STAT4;RORA;PTGS2
**R-HSA-8935964**	RUNX1 regulates expression of components of tight junctions	0.0022	0.1007	CLDN5
**R-HSA-156590**	Glutathione conjugation	0.0027	0.1007	HPGDS;GSTM1
**R-HSA-446652**	Interleukin-1 family signaling	0.0028	0.1007	IL1R1;ALOX5;IL18
**R-HSA-9020958**	Interleukin-21 signaling	0.0049	0.1557	STAT4
**R-HSA-913531**	Interferon Signaling	0.0066	0.1928	STAT4;HLA-DPB1;IRF5;SAMHD1;HLA-DPA1
**R-HSA-380108**	Chemokine receptors bind chemokines	0.0083	0.2030	CXCL8;CCR6
**R-HSA-8984722**	Interleukin-35 Signalling	0.0085	0.2030	STAT4

Supplementary Table 5. HOXB mRNA expression values for the HOXB "high" patient subgroup

mRNA expression values (FPKM) from COG TARGET cases (n=252) with greater than 80th percentile rank and FPKM >1 for HOXB2 or B3 or B4 or B5. Values categorizing within the high subgroup for each gene are highlighted. Data are reanalyzed from NCBI dbGaP accession phs000218/000464[1].

Patient ID	HOXB2	HOXB3	HOXB4	HOXB5	First Event	Event Free Survival (Time in Days)
**SJALL015245**	0.7241	1.6321	1.302	0	0	2932
**SJALL015248**	2.3322	2.286	0.8438	0	0	1939
**SJALL015260**	4.7672	0.7558	1.2805	0	0	3003
**SJALL015261**	4.2975	0.8235	2.1856	0	0	2995
**SJALL015263**	4.6286	3.0254	1.0917	0	0	2989
**SJALL015264**	0.099	1.2037	1.313	0	1	1300
**SJALL015267**	3.0569	1.4251	1.8768	0	0	2247
**SJALL015272**	2.6326	1.6002	0.5976	0	0	2995
**SJALL015274**	0.0219	0.595	1.2918	0	0	2443
**SJALL015275**	1.9086	1.6238	1.4765	0	1	110
**SJALL015278**	1.0881	0.3338	1.3078	0	0	3158
**SJALL015281**	5.3149	1.842	1.7475	0	0	3086
**SJALL015282**	8.4286	5.031	0.7999	0	0	2769
**SJALL015586**	0.0188	1.4063	1.2307	0	0	3399
**SJALL015622**	5.1409	2.1087	2.7936	0	1	149
**SJALL015640**	13.7776	8.7685	1.2714	0	0	2403
**SJALL015643**	12.006	7.1978	1.2355	0	0	2238
**SJALL015644**	5.8083	1.1979	1.4931	0	0	2306
**SJALL015645**	3.6969	2.4862	2.5003	0	0	2426
**SJALL015647**	2.5823	1.4901	1.2203	0.0164	0	2126
**SJALL015648**	3.9945	1.055	1.5849	0	0	2321
**SJALL015655**	2.1387	0.8363	1.5712	0	0	2324
**SJALL015658**	2.079	1.143	1.5608	0	1	440
**SJALL015659**	1.6982	1.2219	1.3344	0	1	425
**SJALL015661**	0.3495	0.7589	1.2761	0.0212	0	2006
**SJALL015669**	1.7718	1.724	0.1842	0	0	2167
**SJALL015670**	6.5121	0.9294	1.8997	0	1	191
**SJALL015671**	3.2649	0.6898	0.8434	0	0	2083
**SJALL015672**	6.4878	2.3641	4.01	0	0	2162
**SJALL015673**	3.5121	0.9481	1.3424	0	0	2086
**SJALL015675**	0.095	0.3339	1.3002	0	1	2055
**SJALL015687**	7.7403	1.3213	1.3882	0.0203	0	2055
**SJALL015696**	3.6361	0.4829	0.9363	0	0	1865
**SJALL015701**	4.874	0.553	1.0852	0	0	1832
**SJALL015704**	2.8044	1.8431	0.8774	0	0	1882
**SJALL015705**	3.0061	0.7809	1.5932	0.016	0	1903
**SJALL015706**	5.1585	1.1931	1.7151	0	0	1854
**SJALL015709**	8.2839	0.1551	0.3421	0	0	1870
**SJALL015713**	3.8205	0.7349	1.7235	0	0	1747
**SJALL015714**	0.0936	1.1731	1.2934	0.0265	0	1867
**SJALL015989**	1.8025	1.2702	0.7685	0	1	357
**SJALL015992**	3.298	1.4034	0.625	0	0	2334
**SJALL015993**	3.1858	0.7275	0.8201	0	0	2183
**SJALL015994**	4.5818	7.5528	4.0936	1.4298	1	1166
**SJALL015996**	4.4032	5.4257	3.3025	0	0	2339

**SJALL016003**	2.5617	0.5016	1.2244	0	0	2178
**SJALL016429**	1.0517	1.8301	1.6798	0	0	2841
**SJALL016436**	0.208	0.7416	1.623	0	0	2070
**SJALL016437**	3.7507	1.7889	2.015	0	0	2370
**SJALL016445**	1.1796	1.6722	1.6965	0	0	1955
**SJALL016653**	1.3358	0.7263	1.3172	0	0	2825
**SJBALL001653**	5.2949	1.5056	0.8127	0	0	2313
**SJTALL001636**	14.8229	12.161	11.4393	0.5089	0	2440
**SJTALL001677**	7.0182	2.1863	1.9446	0	0	2340
**SJTALL002005**	4.9066	1.2466	0.9152	0	0	1687
**SJTALL002019**	2.7894	2.8477	1.0441	0	0	3058
**SJTALL002035**	2.0346	2.6507	1.0549	0	0	2432
**SJTALL002036**	2.49	2.893	1.7156	0	0	2752
**SJTALL002049**	2.5779	1.831	0.6137	0	0	2652
**SJTALL002055**	2.5068	1.1568	1.7571	0	0	2359
**SJTALL002075**	7.6001	1.6229	2.3331	0.14	0	2252
**SJTALL002080**	3.5875	1.2202	1.6372	0	0	2253
**SJTALL002089**	5.4119	8.1788	3.6829	0.8525	0	87
**SJTALL002105**	4.0716	1.028	0.4911	0	0	2025
**SJTALL002109**	3.5022	0.7102	1.4094	0	0	1469
**SJTALL002110**	2.562	0.7844	0.4865	0	1	128
**SJTALL002151**	0.9234	1.2353	0.9046	0	0	1854
**SJTALL002258**	1.1093	1.7107	0.8959	0	0	821
**SJTALL021648**	2.773	1.7805	1.1082	0.0296	0	2956
**SJTALL021672**	10.2645	8.1433	7.1024	0.0155	0	2325
**SJTALL021689**	12.4201	18.6501	10.7519	2.5617	1	469
**SJTALL022098**	2.9023	1.2231	1.1455	0	0	2626
**SJTALL022100**	2.8934	2.8541	2.567	0	1	188
**SJTALL022118**	2.9585	2.5481	5.9288	0	0	2125
**SJTALL022447**	8.8447	15.6342	10.3247	9.7558	0	235

Supplementary Table 6. shRNA clones targeting HOX genes and non-targeting controls

Clones were obtained from the RNAi Consortium (TRC), Addgene, and Sigma-Aldrich (SHC).

Gene Symbol	Clone ID	Sequence
HOXA5	TRCN0000012521	CCGGACTACCAGTTGCATAAT
HOXA5	TRCN0000012522	CCTCTCCGAGAGACAAATTAA
HOXA5	TRCN0000017528	GCCATTATAGCGCCTGTATAA
HOXA5	TRCN0000017529	CCGCAGAAGGAGGATTGAAAT
HOXA5	TRCN0000017530	GCTGCACATAAGTCATGACAA
HOXA5	TRCN0000017531	CAGTTGCATAATTATGGAGAT
HOXA7	TRCN0000012503	GAAGTGGAAGAAAGAGCATAA
HOXA7	TRCN0000012504	CGGGCTTATACAATGTCAACA
HOXA7	TRCN0000012506	CGAGCCGACTTCTTGCTCCTT
HOXA7	TRCN0000015083	CTTTAAGAGACTCACTGGTTT
HOXA7	TRCN0000015084	TCCGGGCTTATACAATGTCAA
HOXA7	TRCN0000015085	GTGGAAGAAAGAGCATAAGGA
HOXA7	TRCN0000015086	CCTCGACCGTTCCGGGCTTAT
HOXA7	TRCN0000015087	GATGCGGTCTTCAGGACCTGA
HOXA7	TRCN0000240652	TCTGGTTCCAGAACCGCCGAA
HOXA9	TRCN0000012509	CACGCTTGACACTCACACTTT
HOXA9	TRCN0000012510	GTGGTTCTCCTCCAGTTGATA
HOXA9	TRCN0000012512	GCATTAAACCTGAACCGCTCT
HOXA9	TRCN0000015163	CGGGCATTTAAGTCTGTCCAT
HOXA9	TRCN0000015164	CGCCTCGTGGAACCCAGTGCA
HOXA9	TRCN0000015166	CGCTGTACCCGCTGCGGTGTA
HOXA9	TRCN0000015167	CAGTCCAAGGCGACGGTGTTT
HOXA9	TRCN0000271293	TGGTTCTCCTCCAGTTGATAG
HOXA9	TRCN0000271295	TGCTGATTGTAACGGAGTTAA
HOXA9	TRCN0000284361	GATGCCATTTGGGCTTATTTA
HOXA10	TRCN0000012515	CGCAGGATGAAACTGAAGAAA
HOXA10	TRCN0000015248	CCCTATCTTGTGAAGTTGTTT
HOXA10	TRCN0000015249	CGCGCAGAACATCAAAGAAGA
HOXA10	TRCN0000015250	CACGGCAAAGAGTGGTCGGAA
HOXA10	TRCN0000015251	TCGCCCATAGACCTGTGGCTA
HOXA10	TRCN0000015252	CTCACGGACAGACAAGTGAAA
HOXA10	TRCN0000096008	TGGTTTCAGAACCGCAGGAAT
HOXB2	TRCN0000015558	CCAATGAAATCTCAGGAATAA
HOXB2	TRCN0000015559	CCGCCAAGAAACCCAGCCAAT
HOXB2	TRCN0000015560	CGGCCTTTAGCCGTTCGCTTA
HOXB2	TRCN0000015561	CCCGCTGTCTTGGAGACATTT
HOXB2	TRCN0000015562	CTTGGATGAAAGAGAAGAAAT

HOXB2	TRCN0000329775	TTACTGAATTAGCGTTTAATC
HOXB3	TRCN0000015643	CGGAAAGGAATCCACATCATA
HOXB3	TRCN0000015644	GCCACTAGCAACAGCAGTAAT
HOXB3	TRCN0000015645	GCCGGCTTCATGAACGCCTTA
HOXB3	TRCN0000015646	GCTGCTCTCTTCGGAGGCTAT
HOXB3	TRCN0000015647	GAATCCAAGAAGCGCCCAAAT
HOXB3	TRCN0000070845	CACCCTCACCAAACAGATATT
HOXB4	TRCN0000015713	CCCACACTTTATATACGAATA
HOXB4	TRCN0000015714	AGGAATATTCACAGAGCGATT
HOXB4	TRCN0000015715	AGTTCACGTGAGCACGGTAAA
HOXB4	TRCN0000015716	CCGTCCCACTCCGCGTGCAAA
HOXB4	TRCN0000015717	CGATTACCTACCCAGCGACCA
HOXB5	TRCN0000015793	CGCCAGTGTTTCTGAATGTTT
HOXB5	TRCN0000015794	CCGGACTATCAGTTGCTAAAT
HOXB5	TRCN0000015795	GCTTCACATCAGCCATGATAT
HOXB5	TRCN0000015796	CGGCTACAATTACAATGGGAT
HOXB5	TRCN0000015797	CAGCGCCAATTTCACCGAAAT
HOXB5	TRCN0000054869	CCGGCTCTTACGGCTACAATT
HOXB5	TRCN0000054872	CAGCTCTCTGAGCGGCTCTTA
control (Scramble)	Addgene #1864	CCTAAGGTTAAGTCGCCCTCG
control (Luciferase)	SHC007	CGCTGAGTACTTCGAAATGTC
control (Empty Vector)	SHC001	none

Supplementary Table 7. List of oligonucleotide primers used for NGS library construction

Primer Name	Primer Sequence
P5_ORF Stag 0a	AAT GAT ACG GCG ACC ACC GAG ATC TAC ACT CTT TCC CTA CAC GAC GCT CTT CCG ATC TTC TTG TGG AAA GGA CGA
P5_ORF Stag 1 A	AAT GAT ACG GCG ACC ACC GAG ATC TAC ACT CTT TCC CTA CAC GAC GCT CTT CCG ATC TAT CTT GTG GAA AGG ACG A
P5_ORF Stag 2 GA	AAT GAT ACG GCG ACC ACC GAG ATC TAC ACT CTT TCC CTA CAC GAC GCT CTT CCG ATC TGA TCT TGT GGA AAG GAC GA
P5_ORF Stag 3 CGA	AAT GAT ACG GCG ACC ACC GAG ATC TAC ACT CTT TCC CTA CAC GAC GCT CTT CCG ATC TCG ATC TTG TGG AAA GGA CGA
P5_ORF Stag 4 ACGA	AAT GAT ACG GCG ACC ACC GAG ATC TAC ACT CTT TCC CTA CAC GAC GCT CTT CCG ATC TAC GAT CTT GTG GAA AGG ACG A
P5_ORF Stag 6 CTAGAA	AAT GAT ACG GCG ACC ACC GAG ATC TAC ACT CTT TCC CTA CAC GAC GCT CTT CCG ATC TCT AGA ATC TTG TGG AAA GGA CGA
P5_ORF Stag 7 GACGACA	AAT GAT ACG GCG ACC ACC GAG ATC TAC ACT CTT TCC CTA CAC GAC GCT CTT CCG ATC TGA CGA CAT CTT GTG GAA AGG ACG A
P5_ORF Stag 8 TGGACACA	AAT GAT ACG GCG ACC ACC GAG ATC TAC ACT CTT TCC CTA CAC GAC GCT CTT CCG ATC TTG GAC ACA TCT TGT GGA AAG GAC GA
P7_IND A01	CAA GCA GAA GAC GGC ATA CGA GAT CGG TTC AAG TGA CTG GAG TTC AGA CGT GTG CTC TTC CGA TCT TCT ACT ATT CTT TCC CCT GCA CTG T
P7_IND A02	CAA GCA GAA GAC GGC ATA CGA GAT GCT GGA TTG TGA CTG GAG TTC AGA CGT GTG CTC TTC CGA TCT TCT ACT ATT CTT TCC CCT GCA CTG T
P7_IND A03	CAA GCA GAA GAC GGC ATA CGA GAT TAA CTC GGG TGA CTG GAG TTC AGA CGT GTG CTC TTC CGA TCT TCT ACT ATT CTT TCC CCT GCA CTG T
P7_IND A04	CAA GCA GAA GAC GGC ATA CGA GAT TAA CAG TTG TGA CTG GAG TTC AGA CGT GTG CTC TTC CGA TCT TCT ACT ATT CTT TCC CCT GCA CTG T
P7_IND A05	CAA GCA GAA GAC GGC ATA CGA GAT ATA CTC AAG TGA CTG GAG TTC AGA CGT GTG CTC TTC CGA TCT TCT ACT ATT CTT TCC CCT GCA CTG T
P7_IND A06	CAA GCA GAA GAC GGC ATA CGA GAT GCT GAG AAG TGA CTG GAG TTC AGA CGT GTG CTC TTC CGA TCT TCT ACT ATT CTT TCC CCT GCA CTG T
P7_IND A07	CAA GCA GAA GAC GGC ATA CGA GAT ATT GGA GGG TGA CTG GAG TTC AGA CGT GTG CTC TTC CGA TCT TCT ACT ATT CTT TCC CCT GCA CTG T
P7_IND A08	CAA GCA GAA GAC GGC ATA CGA GAT TAG TCT AAG TGA CTG GAG TTC AGA CGT GTG CTC TTC CGA TCT TCT ACT ATT CTT TCC CCT GCA CTG T
P7_IND A09	CAA GCA GAA GAC GGC ATA CGA GAT CGG TGA CCG TGA CTG GAG TTC AGA CGT GTG CTC TTC CGA TCT TCT ACT ATT CTT TCC CCT GCA CTG T
P7_IND A10	CAA GCA GAA GAC GGC ATA CGA GAT TAC AGA GGG TGA CTG GAG TTC AGA CGT GTG CTC TTC CGA TCT TCT ACT ATT CTT TCC CCT GCA CTG T
P7_IND A11	CAA GCA GAA GAC GGC ATA CGA GAT ATT GTC AAG TGA CTG GAG TTC AGA CGT GTG CTC TTC CGA TCT TCT ACT ATT CTT TCC CCT GCA CTG T
P7_IND A12	CAA GCA GAA GAC GGC ATA CGA GAT TAT GTC TTG TGA CTG GAG TTC AGA CGT GTG CTC TTC CGA TCT TCT ACT ATT CTT TCC CCT GCA CTG T
P7_IND B01	CAA GCA GAA GAC GGC ATA CGA GAT ATT GGA TTG TGA CTG GAG TTC AGA CGT GTG CTC TTC CGA TCT TCT ACT ATT CTT TCC CCT GCA CTG T
P7_IND B02	CAA GCA GAA GAC GGC ATA CGA GAT ATA CTC GGG TGA CTG GAG TTC AGA CGT GTG CTC TTC CGA TCT TCT ACT ATT CTT TCC CCT GCA CTG T
P7_IND B03	CAA GCA GAA GAC GGC ATA CGA GAT TAT GAG AAG TGA CTG GAG TTC AGA CGT GTG CTC TTC CGA TCT TCT ACT ATT CTT TCC CCT GCA CTG T

P7_IND B04	CAA GCA GAA GAC GGC ATA CGA GAT GCA CAG TTG TGA CTG GAG TTC AGA CGT GTG CTC TTC CGA TCT TCT ACT ATT CTT TCC CCT GCA CTG T
P7_IND B05	CAA GCA GAA GAC GGC ATA CGA GAT CGT GGA TTG TGA CTG GAG TTC AGA CGT GTG CTC TTC CGA TCT TCT ACT ATT CTT TCC CCT GCA CTG T
P7_IND B06	CAA GCA GAA GAC GGC ATA CGA GAT TAG TAG AAG TGA CTG GAG TTC AGA CGT GTG CTC TTC CGA TCT TCT ACT ATT CTT TCC CCT GCA CTG T
P7_IND B07	CAA GCA GAA GAC GGC ATA CGA GAT GCA CGA TTG TGA CTG GAG TTC AGA CGT GTG CTC TTC CGA TCT TCT ACT ATT CTT TCC CCT GCA CTG T
P7_IND B08	CAA GCA GAA GAC GGC ATA CGA GAT CGG TAG CCG TGA CTG GAG TTC AGA CGT GTG CTC TTC CGA TCT TCT ACT ATT CTT TCC CCT GCA CTG T
P7_IND B09	CAA GCA GAA GAC GGC ATA CGA GAT TAG TTC TTG TGA CTG GAG TTC AGA CGT GTG CTC TTC CGA TCT TCT ACT ATT CTT TCC CCT GCA CTG T
P7_IND B10	CAA GCA GAA GAC GGC ATA CGA GAT TAC AAG TTG TGA CTG GAG TTC AGA CGT GTG CTC TTC CGA TCT TCT ACT ATT CTT TCC CCT GCA CTG T
P7_IND B11	CAA GCA GAA GAC GGC ATA CGA GAT ATC ACT GGG TGA CTG GAG TTC AGA CGT GTG CTC TTC CGA TCT TCT ACT ATT CTT TCC CCT GCA CTG T
P7_IND B12	CAA GCA GAA GAC GGC ATA CGA GAT CGC ATC AAG TGA CTG GAG TTC AGA CGT GTG CTC TTC CGA TCT TCT ACT ATT CTT TCC CCT GCA CTG T

Supplementary References

1. Liu Y*, et al.* The genomic landscape of pediatric and young adult T-lineage acute lymphoblastic leukemia. *Nat Genet* **49**, 1211-1218 (2017).

2. Townsend Elizabeth C*, et al.* The Public Repository of Xenografts Enables Discovery and Randomized Phase II-like Trials in Mice. *Cancer Cell* **29**, 574-586 (2016).

3. Kalender Atak Z*, et al.* Comprehensive Analysis of Transcriptome Variation Uncovers Known and Novel Driver Events in T-Cell Acute Lymphoblastic Leukemia. *PLoS Genetics* **9**, e1003997 (2013).
