# Supplementary material for: Synthetic modeling reveals HOXB genes are critical for the initiation and maintenance of human leukemia
Source: Nat Commun. 2019 Jul 2;10:2913. doi: 10.1038/s41467-019-10510-8 (PMC6606637; doi:10.1038/s41467-019-10510-8)
Supplement: Supplementary file 3 — Description of Additional Supplementary Files [file 41467_2019_10510_MOESM3_ESM.docx]

**Description of Supplementary Files**

**File Name:** Supplementary Data 1

**Description:** *Summary of transduced CB transplantation experiments (primary recipients)* NED, no evidence of disease; DN, CD4- CD8-; DP, CD4+ CD8+; SP8, CD4- CD8dim+; NA, not assessed.

**File Name:** Supplementary Data 2

**Description:** *Summary of CB leukemia serial transplantation experiments (secondary recipients)* NED, no evidence of disease; IV, intravenous.

**File Name:** Supplementary Data 3

**Description:** *Differentially expressed genes in NLTB-transduced vs. non-transduced CB cells* List of differentially expressed genes based on RNA-seq data using log2 fold-change (FC) >1 and FDR <0.1 for the comparison NLTB-transduced (G+C+; 4 samples) vs. non-transduced (G-C-; 4 samples) CB cells cultured for 14 or 24 days. Genes are listed in descending order of log2FC. Log2FC for 243 upregulated genes (G+C+ > G-C-) is highlighted in orange, and for 225 downregulated genes (G+C+ < G-C-) in blue. HOXB genes are highlighted in yellow.

**File Name:** Supplementary Data 4

**Description:** *Differentially expressed genes in NLTB-transduced CB cells in vitro vs. NLTB leukemia cells in vivo* List of differentially expressed genes based on RNA-seq data using log2 fold-change (FC) >2.5 and FDR <0.1 for the comparison NLTB-transduced CB cells from days 14+24 in vitro (G+C+; 4 samples) vs. primary NLTB leukemias (G+C+; 8 samples). Genes are listed in descending order of log2FC. Log2FC for 96 upregulated genes (in vitro > in vivo) is highlighted in orange, and for 31 downregulated genes (in vitro < in vivo) in blue.

**File Name:** Supplementary Data 5

**Description:** Single nucleotide variant (SNV) calls from whole exome sequencing data.

**File Name:** Supplementary Data 6

**Description:** Gene expression values from CB leukemias and PDX samples after batch correction.

**File Name:** Supplementary Data 7

**Description:** Gene expression values from NLTB-transduced CB cells cultured in *vitro.*

**File Name:** Supplementary Data 8

**Description:** Gene expression values from 15 NLTB (G+C+) CB leukemia samples.
