# Supplementary material for: Synthetic modeling reveals HOXB genes are critical for the initiation and maintenance of human leukemia
Source: Nat Commun. 2019 Jul 2;10:2913. doi: 10.1038/s41467-019-10510-8 (PMC6606637; doi:10.1038/s41467-019-10510-8)
Supplement: Supplementary file 12 — Reporting Summary [file 41467_2019_10510_MOESM12_ESM.pdf]

nature research

Corresponding author(s): Andrew P. Weng

Last updated by author(s): May 7, 2019

Reporting Summary

Nature Research wishes to improve the reproducibility of the work that we publish. This form provides structure for consistency and transparency in reporting. For further information on Nature Research policies, see Authors & Referees and the Editorial Policy Checklist.

Statistics

For all statistical analyses, confirm that the following items are present in the figure legend, table legend, main text, or Methods section.

n/a	Confirmed	
☐	☒	The exact sample size (*n*) for each experimental group/condition, given as a discrete number and unit of measurement
☐	☒	A statement on whether measurements were taken from distinct samples or whether the same sample was measured repeatedly
☐	☒	The statistical test(s) used AND whether they are one- or two-sided<br>*Only common tests should be described solely by name; describe more complex techniques in the Methods section.*
☐	☒	A description of all covariates tested
☐	☒	A description of any assumptions or corrections, such as tests of normality and adjustment for multiple comparisons
☐	☒	A full description of the statistical parameters including central tendency (e.g. means) or other basic estimates (e.g. regression coefficient) AND variation (e.g. standard deviation) or associated estimates of uncertainty (e.g. confidence intervals)
☐	☒	For null hypothesis testing, the test statistic (e.g. *F*, *t*, *r*) with confidence intervals, effect sizes, degrees of freedom and *P* value noted<br>*Give P values as exact values whenever suitable.*
☒	☐	For Bayesian analysis, information on the choice of priors and Markov chain Monte Carlo settings
☒	☐	For hierarchical and complex designs, identification of the appropriate level for tests and full reporting of outcomes
☒	☐	Estimates of effect sizes (e.g. Cohen's *d*, Pearson's *r*), indicating how they were calculated

Our web collection on statistics for biologists contains articles on many of the points above.

Software and code

Policy information about availability of computer code

Data collection	No software was used for data collection.
Data analysis	GraphPad Prism v8.0.1, ELDA tool (http://bioinf.wehi.edu.au/software/elda/)

For manuscripts utilizing custom algorithms or software that are central to the research but not yet described in published literature, software must be made available to editors/reviewers. We strongly encourage code deposition in a community repository (e.g. GitHub). See the Nature Research guidelines for submitting code & software for further information.

Data

Policy information about availability of data

All manuscripts must include a data availability statement. This statement should provide the following information, where applicable:

- Accession codes, unique identifiers, or web links for publicly available datasets
- A list of figures that have associated raw data
- A description of any restrictions on data availability

RNA-seq data from 17 of 22 T-ALL, 24 B-ALL, and 24 AML PDX samples referenced during the study are available in the NCBI SRA database under the accession code SRP103099 [https://trace.ncbi.nlm.nih.gov/Traces/sra/sra.cgi?study=SRP103099]. RNA-seq data and associated clinical annotations for samples from the COG TARGET study referenced during the study are available in the database of Genotypes and Phenotypes (dbGaP) under the accession code phs000218/000464 [https://www.ncbi.nlm.nih.gov/projects/gap/cgi-bin/study.cgi?study_id=phs000218.v21.p7]. RNA-seq data for normal hematopoietic progenitors referenced during the study are available in the National Center for Biotechnology Information Gene Expression Omnibus (GEO) database under the accession code GSE69239 [https://www.ncbi.nlm.nih.gov/geo/query/acc.cgi?acc=GSE69239]. RNA-seq data for T-ALL cell lines referenced during the study are available in the European Genome-phenome Archive (EGA) database under the accession code EGAS00001000536 [https://ega-archive.org/studies/EGAS00001000536].
Whole exome sequencing (WES), RNA-seq, and ChIP-seqdata generated during the current study excluding that in Supplementary Figure 9 have been deposited in the EGA database under accession code EGAS00001003627 [https://ega-archive.org/studies/EGAS00001003627]. ChIP-seq peak call (BED) files have been deposited in the GEO database under accession code GSE130743 [https://www.ncbi.nlm.nih.gov/geo/query/acc.cgi?acc=GSE130743]. SNV calls from WES data underlying

Figure 4a are provided as Supplementary Data 5. Gene expression values from Processed RNA-seq data files underlying Figure 4b/8a, Figure 5a/b, and Supplementary Figure 8 are included as Supplementary Data 5-76-8, respectively. SNV calls from WES data underlying Figure 4a are provided as Supplementary Data 8. Data from Supplementary Figure 9 is available from the corresponding author upon reasonable request. All other data supporting the findings of this study are available within the article and its Supplementary Information files, or from the corresponding author upon reasonable request. A reporting summary for this article is available as a Supplementary Information file.

Field-specific reporting

Please select the one below that is the best fit for your research. If you are not sure, read the appropriate sections before making your selection.

☒ Life sciences ☐ Behavioural & social sciences ☐ Ecological, evolutionary & environmental sciences

For a reference copy of the document with all sections, see nature.com/documents/nr-reporting-summary-flat.pdf

Life sciences study design

All studies must disclose on these points even when the disclosure is negative.

Sample size

No sample-size calculation was performed. Sample sizes were chosen to be as large as possible given resource constraints. The sample sizes were deemed sufficient to support conclusions if statistical signficance was met. If the sample sizes were insufficient to support drawing of conclusions, then no conclusions were made.

Data exclusions

No data were excluded from analysis.

Replication

Experiments were replicated as indicated in the associated figure legends and/or manuscript text.

Randomization

Samples were not randomly allocated into experimental groups. Co-variates were controlled by performing isogenic comparisons.

Blinding

Blinding is not relevant to the current study as samples were not randomly allocated to subgroups for analysis.

Reporting for specific materials, systems and methods

We require information from authors about some types of materials, experimental systems and methods used in many studies. Here, indicate whether each material, system or method listed is relevant to your study. If you are not sure if a list item applies to your research, read the appropriate section before selecting a response.

Materials & experimental systems

n/a	Involved in the study	
☐	☒	Antibodies
☐	☒	Eukaryotic cell lines
☒	☐	Palaeontology
☐	☒	Animals and other organisms
☒	☐	Human research participants
☒	☐	Clinical data

Methods

n/a	Involved in the study	
☐	☒	ChIP-seq
☐	☒	Flow cytometry
☒	☐	MRI-based neuroimaging

Antibodies

Antibodies used

Antibodies used for Flow Cytometry:
PE/Cy7 anti-human CD1a, HI149 (BioLegend, Cat# 300122), BV510 anti-human CD2, RPA-2.10 (BioLegend, Cat# 300218), APC-H7 anti-human CD2, RPA-2.10 (BD Biosciences, Cat# 562638), APC/Fire 750 anti-human CD2, TS1/8 (BioLegend, Cat# 309222), BV510 anti-human CD3, UCHT1 (BioLegend, Cat# 300448), BV605 anti-human CD3, HIT3a (BD Biosciences, Cat# 564712), BV605 anti-human CD4, RPA-T4 (BD Biosciences, Cat# 562658), PE anti-human CD7, CD7-6B7 (BioLegend, Cat# 343106), APC anti-human CD8, RPA-T8 (BD Biosciences, Cat# 555369), APC anti-human CD34, 581 (BioLegend, Cat# 343510), PerCP/Cyanine5.5 anti-human CD38, HIT2 (BioLegend, Cat# 303522), AF700 anti-human CD45, HI30 (BioLegend, Cat# 304024), APC anti-human CD271 (NGFR), ME20.4 (Biolegend, Cat# 345108), PerCP/Cy5.5 anti-human CD271 (NGFR), ME20.4 (Biolegend, Cat# 345112), PE anti-human TCF α/β, IP26 (BioLegend, Cat# 306708), PerCP/Cyanine5.5 anti-human TCR γ/δ, B1 (Biolegend, Cat# 331224).

Antibodies used for Western Blot:
anti-FLAG, M2 (MilliporeSigma, Cat# F1804), anti-β-Actin, AC-15 (MilliporeSigma, Cat# A1978).

Antibodies used for ChIP-seq:
anti-H3K27me3, poly (Diagenode, Cat# pAb-069-050 (C15410069), Lot# A1811-001P), anti-H3K27ac, CMA309 (Kimura et al. 2008, PMID: 18227620).

	Antibody used for animal injections:<br>InVivoMab anti-mouse/human IL7, M25 (Bio X Cell, Cat# BE0048).
Validation	All antibodies were validated by the supplier for human samples.

Eukaryotic cell lines

Policy information about cell lines

Cell line source(s)	OP9-DL1 (JC Zuniga-Pflucker), HSB2 (J Aster), PEER (J Aster), PF382 (J Aster), 293T (J Aster)
Authentication	Cell line identities were verified by STR profiling (PowerPlex 16HS; Promega).
Mycoplasma contamination	All cell lines tested negative for mycoplasma contamination (MycoAlert detection kit; Lonza).
Commonly misidentified lines<br>(See ICLAC register)	None.

Animals and other organisms

Policy information about studies involving animals; ARRIVE guidelines recommended for reporting animal research

Laboratory animals	Mus musculus, NOD.Cg-Prkdcscid Il2rgtm1Wjl/SzJ (aka "NSG"), 0-20 weeks of age, males and females.
Wild animals	The study did not involve wild animals.
Field-collected samples	The study did not involve samples collected from the field.
Ethics oversight	Research Ethics Board of the University of British Columbia and Children's & Women's Hospital of BC. University of British Columbia Animal Care Committee.

Note that full information on the approval of the study protocol must also be provided in the manuscript.

ChIP-seq

Data deposition

☒ Confirm that both raw and final processed data have been deposited in a public database such as GEO.

☒ Confirm that you have deposited or provided access to graph files (e.g. BED files) for the called peaks.

Data access links<br>*May remain private before publication.*	EGA accession EGAS00001003627 [https://www.ebi.ac.uk/ega/studies/EGAS00001003627]<br>GEO accession GSE130743 [https://www.ncbi.nlm.nih.gov/geo/query/acc.cgi?acc=GSE130743]
Files in database submission	d14_GFP_neg_Cherry_neg_H3K27ac_1.fastq<br>d14_GFP_neg_Cherry_neg_H3K27ac_2.fastq<br>d14_GFP_pos_Cherry_pos_H3K27ac_1.fastq<br>d14_GFP_pos_Cherry_pos_H3K27ac_2.fastq<br>d24_GFP_neg_Cherry_neg_H3K27ac_1.fastq<br>d24_GFP_neg_Cherry_neg_H3K27ac_2.fastq<br>d24_GFP_pos_Cherry_pos_H3K27ac_1.fastq<br>d24_GFP_pos_Cherry_pos_H3K27ac_2.fastq<br>d14_GFP_neg_Cherry_neg_H3K27me3_1.fastq<br>d14_GFP_neg_Cherry_neg_H3K27me3_2.fastq<br>d14_GFP_pos_Cherry_pos_H3K27me3_1.fastq<br>d14_GFP_pos_Cherry_pos_H3K27me3_2.fastq<br>d24_GFP_neg_Cherry_neg_H3K27me3_1.fastq<br>d24_GFP_neg_Cherry_neg_H3K27me3_2.fastq<br>d24_GFP_pos_Cherry_pos_H3K27me3_1.fastq<br>d24_GFP_pos_Cherry_pos_H3K27me3_2.fastq<br>d47_GFP_neg_Cherry_neg_H3K27me3_1.fastq<br>d47_GFP_neg_Cherry_neg_H3K27me3_2.fastq<br>d47_GFP_pos_Cherry_pos_H3K27me3_1.fastq<br>d47_GFP_pos_Cherry_pos_H3K27me3_2.fastq<br><br>d14_GFP_neg_Cherry_neg_H3K27me3_peaks.broadPeak<br>d14_GFP_pos_Cherry_pos_H3K27me3_peaks.broadPeak<br>d24_GFP_neg_Cherry_neg_H3K27me3_peaks.broadPeak<br>d24_GFP_pos_Cherry_pos_H3K27me3_peaks.broadPeak<br>d47_GFP_neg_Cherry_neg_H3K27me3_peaks.broadPeak<br>d47_GFP_pos_Cherry_pos_H3K27me3_peaks.broadPeak<br>d14_GFP_neg_Cherry_neg_H3K27ac_peaks.narrowPeak<br>d14_GFP_pos_Cherry_pos_H3K27ac_peaks.narrowPeak

d24_GFP_neg_Cherry_neg_H3K27ac_peaks.narrowPeak
d24_GFP_pos_Cherry_pos_H3K27ac_peaks.narrowPeak
d14_GFP_neg_Cherry_neg_H3K27me3_input
d14_GFP_pos_Cherry_pos_H3K27me3_input
d24_GFP_neg_Cherry_neg_H3K27me3_input
d24_GFP_pos_Cherry_pos_H3K27me3_input
d47_GFP_neg_Cherry_neg_H3K27me3_input
d47_GFP_pos_Cherry_pos_H3K27me3_input
d14_GFP_neg_Cherry_neg_H3K27ac_input
d14_GFP_pos_Cherry_pos_H3K27ac_input
d24_GFP_neg_Cherry_neg_H3K27ac_input
d24_GFP_pos_Cherry_pos_H3K27ac_input

Genome browser session (e.g. UCSC)

No longer applicable

Methodology

Replicates

H3K27me3 ChIP-seq was done from 3 separate biological samples for each of 2 cell types studied (3 separate time points from the same experimental trial). H3K27ac ChIP-seq was done from 2 separate biological samples for each of 2 cell types studied (2 separate time points from the same experimental trial). We are unable to generate a statistical measure of inter-sample agreement as samples were taken at different time points.

Sequencing depth

H3K27me3 ChIP-seq libraries were paired-end sequenced with read length of 125bp, yielding 120-148M total reads per sample and 98-113M aligned reads per sample (n=6). H3K27ac ChIP-seq libraries were paired-end sequenced with read length of 125bp, yielding 25-62M total reads per sample and 21-45M aligned reads per sample (n=4).

Antibodies

Anti-H3K27me3 polyclonal antibody: Diagenode Cat# pAb-069-050 (C15410069), Lot# A1811-001P.
Anti-H3K27ac monoclonal antibody: CMA309 (Kimura et al. 2008; PMID: 18227620).

Peak calling parameters

Reads were aligned to NCBI Build 37 (hg19) human reference genome using BWA-backtrack v0.5.7 and default parameters. Peak enrichment was computed using MACS v2.1.1 (https://github.com/taoliu/MACS) with a false discovery rate (FDR) value of 0.1 for H3K27me3 and FDR of 0.01 for H3K27ac. Input sample was used as control background.
H3K27me3: macs2 callpeak -f BAMPE -g hs -t <H3K27me3.bam> -c <input.bam> --qvalue 0.05 --broad --broad-cutoff 0.1
H3K27ac: macs2 callpeak -f BAMPE -g hs -t <H3K27ac.bam> -c <input.bam> -q 0.01

Data quality

A standardized analytical pipeline introduced by Canadian Epigenetics, Environment, and Health Research Consortium [CEEHRC] and International Human Epigenomic Consortium (IHEC) (http://ihec-epigenomes.org/) was applied to qualify the resulting data. Peaks passing FDR of 0.05 were 130,000-260,000 per sample for H3K27me3 (n=6) and 33,000-39,000 per sample for H3K27ac (n=4).

Software

BWA-backtrack v0.5.7 (Li and Durbin, 2010; PMID: 20080505) with default parameters for read alignment.
MACS v2.1.1 (https://github.com/taoliu/MACS) for peak calling of broadPeak and narrowPeak files.
Bedtools (Quinlan and Hall, 2010; PMID: 20110278)

Flow Cytometry

Plots

Confirm that:

☒ The axis labels state the marker and fluorochrome used (e.g. CD4-FITC).

☒ The axis scales are clearly visible. Include numbers along axes only for bottom left plot of group (a 'group' is an analysis of identical markers).

☒ All plots are contour plots with outliers or pseudocolor plots.

☒ A numerical value for number of cells or percentage (with statistics) is provided.

Methodology

Sample preparation

Live single cell suspensions obtained from bone marrow or spleen of leukemic mice, or live single cell suspensions from tissue culture, filtered through 70um nylon mesh.

Instrument

BD LSR-Fortessa, FACSCalibur

Software

FlowJo

Cell population abundance

CD34+ CB cells were FACS sorted to 95-99% purity as assessed by post-sort analysis on the same instrument. G+C+, G+C-, and G-C- populations from transduced CB cells or primary CB leukemias were FACS sorted to >95% purity as assessed by post-sort analysis on the same instrument. G+C+N+ cells sorted at limiting dilution into 96-well plates for the well-initiation assay (Fig 8e/f) could not be assessed for purity after sorting; however, ~90% of assayable wells after 3 weeks' culture were populated by G+C+N+ cells.

Gating strategy	Example gating strategies are provided in Supplementary Information (Supplementary Figures 18 and 19).

☒ Tick this box to confirm that a figure exemplifying the gating strategy is provided in the Supplementary Information.
